# The Effect of Helminth Infections and Their Treatment on Metabolic Outcomes: Results of a Cluster-Randomized Trial

**DOI:** 10.1093/cid/ciz859

**Published:** 2019-08-30

**Authors:** Richard E Sanya, Emily L Webb, Christopher Zziwa, Robert Kizindo, Moses Sewankambo, Josephine Tumusiime, Esther Nakazibwe, Gloria Oduru, Emmanuel Niwagaba, Prossy Kabuubi Nakawungu, Joyce Kabagenyi, Jacent Nassuuna, Bridgious Walusimbi, Irene Andia-Biraro, Alison M Elliott

**Affiliations:** 1 Immunomodulation and Vaccines Programme, Medical Research Council (MRC)/Uganda Virus Research Institute and London School of Hygiene and Tropical Medicine Uganda Research Unit, Entebbe, Uganda; 2 Department of Internal Medicine, College of Health Sciences, Makerere University, Kampala, Uganda; 3 MRC Tropical Epidemiology Group, Department of Infectious Disease Epidemiology, London School of Hygiene and Tropical Medicine, London, United Kingdom; 4 Department of Clinical Research, London School of Hygiene and Tropical Medicine, London, United Kingdom

**Keywords:** helminths, *Schistosoma mansoni*, diabetes, cardiovascular disease, Africa

## Abstract

**Background:**

Helminths may protect against cardiometabolic risk through effects on inflammation and metabolism; their treatment may be detrimental to metabolic outcomes.

**Methods:**

In a cluster-randomized trial in 26 Ugandan fishing communities we investigated effects of community-wide intensive (quarterly single-dose praziquantel, triple-dose albendazole) vs standard (annual single-dose praziquantel, biannual single-dose albendazole) anthelminthic treatment on metabolic outcomes, and observational associations between helminths and metabolic outcomes. The primary outcome, homeostatic model assessment of insulin resistance (HOMA-IR), and secondary outcomes (including blood pressure, fasting blood glucose, lipids) were assessed after 4 years' intervention among individuals aged ≥10 years.

**Results:**

We analyzed 1898 participants. Intensive treatment had no effect on HOMA-IR (adjusted geometric mean ratio, 0.96 [95% confidence interval {CI}, .86–1.07]; *P* = .42) but resulted in higher mean low-density lipoprotein cholesterol (LDL-c) (2.86 vs 2.60 mmol/L; adjusted mean difference, 0.26 [95% CI, −.03 to .56]; *P* = .08). Lower LDL-c levels were associated with *Schistosoma mansoni* (2.37 vs 2.80 mmol/L; −0.25 [95% CI, −.49 to −.02]; *P* = .04) or *Strongyloides* (2.34 vs 2.69 mmol/L; −0.32 [95% CI, −.53 to −.12]; *P* = .003) infection. *Schistosoma mansoni* was associated with lower total cholesterol (4.24 vs 4.64 mmol/L; −0.25 [95% CI, −.44 to −.07]; *P* = .01) and moderate to heavy *S. mansoni* infection with lower triglycerides, LDL-c, and diastolic blood pressure.

**Conclusions:**

Helminth infections improve lipid profiles and may lower blood pressure. Studies to confirm causality and investigate mechanisms may contribute to understanding the epidemiological transition and suggest new approaches to prevent cardiometabolic disease.

**Clinical Trials Registration:**

ISRCTN47196031.

## Teaser

In a cluster-randomized trial, intensive (vs standard) anthelminthic treatment increased low-density lipoprotein cholesterol. In observational analyses, schistosome and *Strongyloides stercoralis* infections were associated with reduced serum lipids, and heavy schistosome infection with lower blood pressure. Helminth elimination may promote cardiovascular disease risk.

Noncommunicable diseases accounted for 72.3% of deaths globally in 2016 [1]. Ischemic heart disease and cerebrovascular disease are the highest contributors to this mortality and morbidity [1]. Metabolic disorders such as type 2 diabetes mellitus (T2D) and dyslipidemia are important cardiovascular disease risk factors. In 2017 an estimated 425 million people had diabetes worldwide and the number is projected to increase—disproportionately in low- and middle-income countries (LMICs)—to 629 million by 2045 [2]. Dyslipidemia, which results in atherosclerosis, is the leading risk factor for myocardial infarction and stroke [3, 4].

Chronic, low-grade, obesity-driven inflammation has been implicated in the etiology and progression of insulin resistance and T2D [5]. Atherosclerosis results from a chronic inflammatory state mediated by macrophages in the vascular subendothelium combined with high levels of lipids in the systemic circulation [6]. Lipids are utilized by and involved in mediation of immune processes; therefore, inflammation may influence lipid levels in the circulation [7]. Modulation of these inflammatory processes may delay the development of T2D and atherosclerosis.

More than a billion people are helminth-infected worldwide, predominantly in LMICs [8]. Helminths have coevolved with humans over millions of years so that they survive in the human host but mostly cause only subtle harm. To ensure survival, helminths modify type 2 and regulatory immune responses in the human host [9]. With these immunomodulatory properties, helminths may be able to annul the inflammation that results in metabolic disorders and therefore confer protection [10]. Studies in experimental animals support this hypothesis. Mice infected with *Schistosoma mansoni* [11], *Nippostrongylus brasiliensis* [12], and *Litomosoides sigmodontis* [13] had decreased insulin resistance and improved glucose tolerance compared with uninfected mice. Infection of mice with *S. mansoni* cercariae [14] and intraperitoneal inoculation of *S. mansoni* eggs [15] resulted in lower blood lipid levels than in uninfected/uninoculated controls. In mice, *S. mansoni* infection also reduced development of atherosclerotic lesions [14], and ES-62, a product from the filarial nematode *Acanthocheilonema vitae*, caused a 60% reduction in aortic atherosclerotic lesions [16].

In humans, published studies investigating the effect of helminths on metabolic outcomes are few and observational, except for a single trial. In China, history of *Schistosoma japonicum* infection was associated with lower levels of fasting blood glucose, glycated hemoglobin (HbA1c), and insulin resistance, and lower prevalence of diabetes and metabolic syndrome [17], whereas chronic infection with this helminth was associated with lower serum triglycerides and total cholesterol [18]. Among Australian Aboriginals, *Strongyloides stercoralis* infection was associated with a lower likelihood of diabetes [19], and treatment of *Strongyloides* worsened glycemic status [20]. Among the Tsimane of the Bolivian Amazon, helminth infection was inversely associated with serum lipid levels [21]. Chronic infection with the trematode *Opisthorchis felineus* was associated with lower serum cholesterol levels and diminished atherosclerosis in a postmortem study in Russia [22]. In Indonesia, infection with soil-transmitted helminths (STHs) was associated with lower insulin resistance and body mass index (BMI) [23]. The only trial to date, conducted in Indonesia, showed no effect of albendazole treatment for STHs on insulin resistance, BMI, or waist circumference overall, but showed increased insulin resistance in individuals infected with STHs at baseline [24].

Observational studies cannot demonstrate causality and are prone to bias from unmeasured confounders. The only previous trial was done in a setting with high prevalence of STH. However, other parasitic infections such as *S. mansoni* may have a different impact on metabolic outcomes because of the different location of adult worms and inflammatory and regulatory elements of their life cycles. We therefore aimed to investigate, in a setting of high *S. mansoni* prevalence, the effect of helminths and their treatment on metabolic outcomes in individuals aged ≥10 years. We also conducted observational analyses to assess cross-sectional associations between helminths and metabolic outcomes.

## METHODS

### Study Setting and Design

This was a 2-arm, cluster-randomized trial of community-wide intensive vs standard anthelminthic treatment conducted in 26 Lake Victoria fishing villages of Koome subcounty, Mukono district, Uganda (ISRCTN47196031). This population is ethnically diverse with the majority from central Uganda (of Bantu ethnic origin). There is some acculturation as different ethnic groups live together and use Luganda as the lingua franca. The main economic activity is fishing with a minority involved in agriculture. Villages are well defined, geographically separate, and close to the lake. The residents live in temporary housing made from wood (locally obtained) and roofed with iron sheets or plastic. Houses are built close together. The island communities trade with mainland communities (both nearby and far) in fish and general merchandise.

The protocol has been published elsewhere [25]. In brief, 13 villages were randomized to intensive and 13 to standard intervention. Intensive treatment comprised praziquantel 40 mg/kg single-dose administered using an extended height pole (which extended treatment to include preschool age groups) and albendazole 400 mg triple-dose, both quarterly. Standard treatment comprised single-dose praziquantel (40 mg/kg) annually and single-dose albendazole (400 mg) biannually. Trial interventions started in September 2012. Originally, the trial was designed to investigate the effects of 3 years’ intervention on allergy-related outcomes and helminth-related morbidity, but interventions were extended for an extra year, allowing us to assess their impact on metabolic outcomes.

### Randomization and Masking

This was an open trial. Villages were randomized in a 1:1 ratio using restricted randomization to ensure balance for village size, previous community-wide anthelminthic treatment, and distance from the subcounty health center [25].

### Exposures and Outcomes

The exposures of interest were anthelminthic intervention (for the trial) and helminth infections (for observational analyses). The primary outcome was insulin resistance measured by the homeostatic model assessment of insulin resistance (HOMA-IR) [26], calculated as (fasting serum insulin [μU/mL] × fasting glucose [mmol/L]) / 22.5. Secondary outcomes were fasting blood glucose, HbA1c, fasting serum lipids (triglycerides, total cholesterol, high-density lipoprotein cholesterol [HDL-c], low-density lipoprotein cholesterol [LDL-c]), blood pressure, BMI, waist circumference, waist-to-hip ratio, and (for the trial) helminth infection status.

### Procedures

The effects of helminths and anthelminthic intervention were assessed in a household survey conducted in all study villages after 4 years of intervention. We selected 70 households per village by simple random sampling using Stata version 13.0 software (StataCorp, College Station, Texas) (Supplementary Methods). Permission for household participation was sought from each household head or another adult in the household if the head was absent. Household members aged ≥10 years were invited to participate.

After obtaining written informed consent from each household member (and assent from individuals aged 10–17 years), a questionnaire was administered. Data were collected on sociodemographic characteristics, diet, exercise, lifestyle, and personal and family history of diabetes and hypertension. Participants’ blood pressure (BP), weight, height, and waist and hip circumference were measured using standardized procedures (Supplementary Methods). Peripheral venous blood was collected after an overnight fast for measurement of the primary and secondary outcomes (Supplementary Methods).

Each participant was requested to provide 1 stool sample, from which duplicate slides were made and examined independently by 2 experienced technicians using the Kato-Katz method. Stool real-time polymerase chain reaction (PCR) was also used to detect *S. mansoni*, hookworm (*Necator americanus*), and *S. stercoralis* infection (Supplementary Methods).

### Statistical Methods

For this survey, we planned to recruit 1950 participants (Supplementary Methods). Statistical analyses were done using Stata version 13.0 software; all analyses allowed for within-cluster correlations. The trial analysis was by intention-to-treat and measured cluster-level differences. For continuous outcomes, means were calculated for each village (cluster-specific means) and the mean of these within each study arm was used as a summary measure of the outcome in that study arm. The *t* test was used to compare cluster-specific means between arms, with corresponding 95% confidence intervals (CIs) computed. Continuous outcomes with skewed cluster-specific mean distributions were log-transformed before analysis and results back-transformed to give geometric mean ratios. For binary outcomes, we calculated risk ratios by dividing the mean of the cluster-specific proportions in the intensive arm by that in the standard arm. *P* values were calculated from *t* tests comparing cluster-specific proportions, and CIs using a Taylor series approximation to estimate standard errors. For all outcomes, the effects of the intervention were additionally adjusted for age and sex using a 2-stage approach [27].

The individual level observational analysis was performed to assess helminths as risk factors for metabolic disease. Potential confounders were identified based on a causal diagram (Supplementary Figure 1). Crude and adjusted associations were estimated using linear regression models fitted for each worm separately. Stata “svy” commands were used to allow for clustering of participants within villages, and for the non-self-weighting survey design due to variable village sizes. For adjusted analyses, only risk factors/confounders crudely associated with the outcome with *P* value ≤ .15 were included in final models. No corrections were made for multiple testing.

### Ethical Considerations

Ethical clearance was granted by the Uganda Virus Research Institute Research ethics committee (reference GC/127/17/01/573), the London School of Hygiene and Tropical Medicine (reference 9917), and the Uganda National Council for Science and Technology (reference HS 2185). Permission to conduct the work was granted by community leaders in all study villages.

## RESULTS

**Figure 1.**
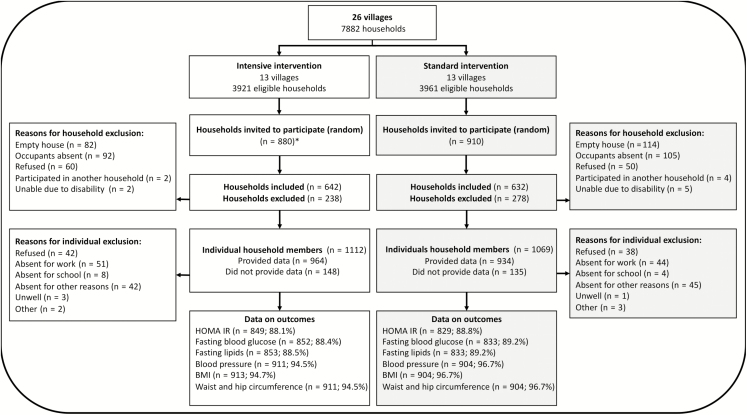
Flowchart of the survey on metabolic outcomes in the Lake Victoria Island Intervention Study on Worms and Allergy-related Diseases cluster-randomized trial. *In 2 villages, the total number of households per village was <70, and therefore all households (41 and 69, respectively) were invited to participate. Abbreviations: BMI, body mass index; HOMA-IR, homeostatic model assessment of insulin resistance.

Between April and November 2017, 70 households were randomly selected from each village except in 2 villages with fewer households where all were selected. Of eligible households and eligible individuals in those households, 71.3% (1276/1790) and 87.0% (1898/2181), respectively, agreed to participate. We examined 1817 of 1898 (95.7%) participants, and 1686 of 1898 (88.8%) provided blood samples after an overnight fast. Numbers of participants providing data were balanced across trial arms (Figure 1).

Characteristics of participants are shown in Table 1. This is a young population with a mean age of 31.5 years (standard deviation [SD], 11 years) considering that individuals aged <10 years were excluded. There are more males than females and the main occupation is fishing; the majority (1309/1898 [69%]) reported daily contact with the lake. Activity levels were high: 920/1898 (48.4%) reported vigorous physical activity at least weekly, with a mean number of days of 2.8 (SD, 2.7 days). Diet mainly consists of fish (eaten on average 4.8 days a week) and is low in fruit (on average 1 day a week). The majority of participants (1218/1898 [64.2%]) reported having lived in the village in which they were surveyed throughout the 4-year intervention period. Only 283 of 1898 (14.9%) had ever undergone BP measurement and 80 of 1898 (4.2%) had previously had blood sugar levels tested.

**Table 1.** Characteristics of Participants Aged ≥10 Years Enrolled in the Survey on Metabolic Outcomes in the Lake Victoria Island Intervention Study on Worms and Allergy-Related Diseases Cluster-Randomized Trial

Cluster-level Characteristics	Study Arm	
	Intensive	Standard
Household-level characteristics		
Household size, median (IQR)	2 (1–3)	2 (1–3)
Individual-level characteristics	(n = 964)	(n = 934)
Sex, male	516 (53.5)	494 (52.9)
Age, y, mean (SD)	32 (11.0)	31 (11.0)
Age group, y		
10–19	105 (10.9)	110 (11.8)
20–29	324 (33.6)	324 (34.7)
30–39	311 (32.3)	283 (30.3)
≥40	224 (23.2)	217 (23.2)
Occupation		
Child/student	50 (5.2)	71 (7.6)
Housewife	103 (10.7)	96 (10.3)
Fishing or lake related	366 (38.0)	365 (39.1)
Shops, salons, artisans, service providers	68 (7.1)	95 (10.2)
Bars, restaurants, food providers, entertainment	70 (7.3)	86 (9.2)
Agriculture, lumbering, charcoal	225 (23.3)	167 (17.8)
Professional	20 (2.1)	11 (1.2)
Unemployed	18 (1.9)	18 (1.9)
Other	44 (4.6)	25 (2.7)
Residence		
Always lived in the village	84 (8.7)	80 (8.6)
Has lived only in the village throughout the intervention period	526 (54.6)	528 (56.3)
Has lived elsewhere in the subcounty during the intervention period	32 (3.2)	41 (4.4)
Lived outside the study area during the intervention period	322 (33.4)	285 (30.5)
Place of birth (if participant has not always lived in the village)		
Fishing village	28 (2.9)	24 (2.6)
Other rural village	757 (78.5)	743 (79.6)
Town	76 (7.9)	65 (7.0)
City	19 (2.0)	22 (2.4)
First 5 years (if participant has not always lived in the village) (n = 13 missing)		
This village	4 (0.4)	8 (0.8)
A fishing village	27 (2.8)	24 (2.6)
Other rural village	747 (77.5)	733 (78.5)
Town	78 (8.1)	66 (7.1)
City	19 (2.0)	22 (2.4)
Age of participant when he/she moved to this village, mean (SD)	24 (11.2)	23 (11.3)
Maternal tribe (grouped by region)		
Central	342 (35.5)	336 (36.0)
Western	156 (16.3)	138 (14.8)
Eastern	215 (22.3)	190 (20.3)
Northern	90 (9.3)	112 (12.0)
Non-Ugandan	157 (16.3)	154 (16.5)
Do not know	4 (0.4)	4 (0.4)
Paternal tribe (grouped by region)		
Central	387 (40.2)	364 (39.0)
Western	165 (17.1)	163 (17.5)
Eastern	213 (22.1)	172 (18.4)
Northern	93 (9.7)	108 (11.6)
Non-Ugandan	105 (10.9)	125 (13.4)
Do not know	1 (0.1)	2 (0.2)
Self-reported treatment for worms		
Ever treated for worms	866 (89.8)	795 (85.1)
No. of times treated with albendazole in the last 12 mo, mean (SD)	1 (0.8)	2 (1.4)
No. of times treated with albendazole in the last 4 y, mean (SD)	8 (5.4)	4 (2.8)
No. of times treated with praziquantel in the last 12 mo, mean (SD)	2 (1.4)	0.5 (0.6)
No. of times treated with praziquantel in the last 4 y, mean (SD)	8 (5.4)	2 (1.6)
Frequency of lake contact (mv, 41)		
Every day	630 (65.3)	679 (72.7)
Almost every day	161 (16.7)	136 (14.6)
Once a week	120 (12.5)	72 (7.7)
Once a month	33 (3.4)	19 (2.0)
Less than once a month	3 (0.3)	4 (0.4)
Ever had blood sugar measured (mv, 30)		
Yes	43 (4.5)	37 (4.0)
No	905 (93.8)	879 (94.1)
Do not know	2 (0.2)	2 (0.2)
Blood sugar measured in the past 12 mo		
Yes	18 (1.9)	13 (1.4)
No	26 (2.7)	25 (2.7)
History of diabetes (mv, 30)		
Yes	4 (0.4)	3 (0.3)
No	932 (96.7)	909 (97.3)
Do not know	14 (1.5)	6 (0.6)
Ever had blood pressure measured (mv, 30)		
Yes	147 (15.3)	136 (14.6)
No	802 (83.2)	782 (83.4)
Do not know	1 (0.1)	0
Blood pressure measured in the past 12 mo (mv, 30)		
Yes	66 (6.9)	61 (6.5)
No	81 (8.4)	75 (8.0)
History of hypertension (mv, 30)		
Yes	20 (2.1)	24 (2.6)
No	925 (96.0)	890 (95.3)
Do not know	5 (0.5)	4 (0.4)
Frequency of exercise/participation in vigorous physical activity (mv, 30)		
Every day	19 (2.0)	8 (0.9)
Almost every day	193 (20.5)	194 (21.2)
Once a week	305 (32.5)	177 (19.3)
Once a month	305 (18.3)	187 (20.4)
Less than once a month	237 (25.2)	333 (36.4)
No. of days in a week a participant performs vigorous physical activity, mean (SD)	3.0 (2.7)	2.6 (2.7)
Hours spent per day performing vigorous physical activity, mean (SD)	3.4 (3.4)	2.9 (3.4)
Diet in a typical week (mv, 30)		
No. of days you eat fruit, mean (SD)	1.0 (1.5)	0.8 (1.4)
No. of servings of fruit you eat on 1 of those days, mean (SD)	0.5 (0.7)	0.4 (0.7)
No. of days you eat vegetables, mean (SD)	0.8 (1.3)	0.4 (1.0)
No. of days you eat fish, mean (SD)	4.8 (2.3)	4.8 (2.2)
No. of days you eat meat, mean (SD)	0.3 (0.9)	0.4 (1.1)
Type of oil or fat that is most often used for meal preparation in the household or where the participant eats (mv, 30)		
Vegetable oil	845 (87.7)	794 (85.0)
Other oils	14 (1.5)	10 (1.1)
None in particular	23 (2.4)	13 (1.4)
None used	54 (5.6)	78 (8.3)
Do not know	14 (1.5)	23 (2.5)
Ever smoked (either pipe or cigarette) (mv, 30)		
Yes	175 (18.2)	168 (18.0)
No	775 (80.4)	750 (80.3)
Currently smoking tobacco products daily	147 (15.3)	128 (13.7)
Ever taken alcohol (mv, 30)	482 (50.0)	423 (45.3)
Currently drinking alcohol	406 (42.1)	352 (37.7)
Maternal history of diabetes (mv, 30)		
No history	801 (83.1)	764 (81.8)
History of diabetes	30 (3.1)	39 (4.2)
Do not know	119 (12.3)	115 (12.3)
Paternal history of diabetes (mv, 30)		
No history	785 (81.4)	790 (84.6)
History of diabetes	28 (2.9)	25 (2.7)
Do not know	137 (14.2)	103 (11.0)
Maternal history of hypertension (mv, 30)		
No history	688 (71.3)	669 (71.6)
History of hypertension	148 (15.4)	137 (14.7)
Do not know	114 (11.8)	112 (12.0)
Paternal history of hypertension (mv, 30)		
No history	764 (79.3)	775 (83.0)
History of hypertension	52 (5.4)	40 (4.3)
Do not know	134 (1.5)	103 (11.0)
Maternal history of obesity/overweight (mv, 30)		
No history	811 (84.1)	774 (82.9)
History of obesity/overweight	14 (1.5)	14 (1.5)
Do not know	125 (13.0)	130 (13.9)
Paternal history of obesity/overweight (mv, 30)		
No history	805 (83.5)	803 (86.0)
History of obesity/overweight	4 (0.4)	8 (0.9)
Do not know	141 (14.6)	107 (11.5)

Data are presented as no. (%) unless otherwise indicated.

Abbreviations: IQR, interquartile range; mv, missing values; SD, standard deviation.

Participants living in intensive anthelminthic intervention villages had lower *S. mansoni* prevalence than those in standard intervention villages (stool Kato-Katz: intensive 21.9%, standard 35.6%, *P* = .02; stool PCR: intensive 37.3%, standard 56.0%, *P* = .01). Similar effects were observed for hookworm (stool PCR: intensive 2.3%, standard 4.8%, *P* = .03) and *Strongyloides* (stool PCR: intensive 4.8%, standard 9.0%, *P* = .02). Around half of *S. mansoni* infections were of light intensity (intensive 12.2%, standard 17.4%). The intervention had no effect on *Trichuris trichiura* (Table 2).

**Table 2.** Effect of Intensive Versus Standard Anthelminthic Treatment on Helminth Prevalence in the Metabolic Survey of the Lake Victoria Island Intervention Study on Worms and Allergy-Related Diseases Cluster-Randomized Trial: Cluster-Level Analysis (n = 1853)

Outcome	%		Crude RR (95% CI)	*P* Value	Adjusted RR^a^ (95% CI)	*P* Value
	Intensive Arm	Standard Arm				
*Schistosoma mansoni*, stool Kato-Katz	21.9	35.6	0.62 (.41–.92)	.02	0.71 (.54–.93)	.02
*S. mansoni*, stool PCR	37.3	56.0	0.67 (.51–.89)	.007	0.76 (.63–.94)	.01
*S. mansoni* intensity, stool Kato-Katz						
Light	12.2	17.4	…		…	
Moderate	4.9	11.4	…		…	
Heavy	4.8	6.8	…		…	
*Trichuris trichiura*, stool Kato-Katz	7.4	8.1	0.91 (.41–2.02)	.82	0.78 (.36–1.68)	.50
Hookworm, stool PCR	2.3	4.8	0.47 (.22–1.01)	.03	0.48 (.23–1.01)	.03
*Strongyloides stercoralis*, stool PCR	4.8	9.0	0.54 (.34–.86)	.01	0.52 (.31–.87)	.02

Abbreviations: CI, confidence interval; PCR, polymerase chain reaction; RR, risk ratio.

^a^Adjusted for age, sex, and baseline helminth prevalence.

There was no evidence of a difference in HOMA-IR between the study arms (Table 3). However, there was some evidence of a difference in mean LDL-c, higher in the intensive arm (2.86 vs 2.60 mmol/L; adjusted mean difference, 0.26 [95% CI, −.03 to .56]; *P* = .08). No differences were seen between trial arms for the other metabolic parameters (Table 3). Further analysis of the metabolic outcomes categorized as binary (diabetes, impaired fasting glucose, hypertension, obesity, and metabolic syndrome) showed no differences between trial arms (Supplementary Table 1).

**Table 3.** Effect of Intensive Versus Standard Anthelminthic Treatment on Metabolic Outcomes in the Metabolic Survey of the Lake Victoria Island Intervention Study on Worms and Allergy-Related Diseases Cluster-Randomized Trial: Cluster-Level Analysis (n = 1853)

Outcome	Mean		Crude Mean Difference or GMR (95% CI)	*P* Value	Adjusted Mean Difference or GMR (95% CI)^a^	*P* Value
	Intensive	Standard				
HOMA-IR (glucose × insulin / 22.5)	GM, 1.24^b^	GM, 1.30^b^	GMR, 0.96 (.85–1.07)^c^	.43	aGMR, 0.96 (.86–1.07)^c^	.42
Fasting glucose (mmol/L)	4.78	4.76	0.02 (−.16 to .20)	.85	0.01 (−.17 to .19)	.90
Glycated hemoglobin (mmol/mol)	30.72	30.56	0.16 (−2.09 to 2.41)	.88	0.14 (−2.11 to 2.40)	.90
Triglycerides (mmol/L)	GM, 0.98^b^	GM, 0.98^b^	GMR, 1.00 (.94–1.06)^c^	.95	aGMR, 0.97 (.85–1.11)^c^	.63
Total cholesterol (mmol/L)	4.52	4.33	0.19 (−.16 to .54)	.27	0.19 (−.16 to .54)	.28
LDL-c (mmol/L)	2.86	2.60	0.27 (−.03 to .56)	.08	0.26 (−.03 to .56)	.08
HDL-c (mmol/L)	GM, 1.20^b^	GM, 1.20^b^	GMR, 1.01 (.87–1.17)^c^	.94	aGMR, 1.01 (.87–1.17)^c^	.94
Systolic BP (mm Hg)	115.27	116.13	−0.86 (−2.84 to 1.12)	.38	−1.02 (−2.72 to .68)	.22
Diastolic BP (mm Hg)	76.40	76.37	0.03 (−1.25 to 1.31)	.97	−0.12 (−1.34 to 1.10)	.84
Body mass index (kg/m^2^)	23.34	23.34	0.01 (−.50 to .51)	.99	−0.08 (−.58 to .42)	.74
Waist circumference (cm)	80.00	80.04	−0.04 (−1.49 to 1.41)	.95	−0.32 (−1.70 to 1.07)	.64
Waist-to-hip ratio	0.85	0.85	0.00 (.00−.01)	.96	0.00 (−.01 to .00)	.72

Abbreviations: aGMR, adjusted geometric mean ratio; BP, blood pressure; CI, confidence interval; GM, geometric mean; GMR, geometric mean ratio; HDL-c, high-density lipoprotein cholesterol; HOMA-IR, homeostatic model assessment of insulin resistance; LDL-c, low-density lipoprotein cholesterol.

^a^Adjusted for age and sex.

^b^Geometric means used because of skewed cluster-specific means.

^c^GMRs used because of skewed cluster-specific means.

Key associations from our observational analysis are shown in Table 4; all associations tested are shown in Supplementary Table 2. *Schistosoma mansoni*–infected participants had lower total cholesterol levels than uninfected participants (4.24 mmol/L vs 4.64 mmol/L; crude mean difference, −0.40 [95% CI, −.62 to −.19]; *P* = .001). Strong evidence for this association remained after adjusting for age, sex, occupation, residence, diet, exercise, family history of obesity, and paternal and maternal tribe (−0.25 [95% CI, −.44 to .07]; *P* = .01). *Schistosoma mansoni* infection was associated with lower LDL-c (2.37 vs 2.80 mmol/L; −0.25 [95% CI, −.49 to −.02]; *P* = .04) but not with HDL-c. *Strongyloides* infection was associated with lower LDL-c levels (*P* = .003). Participants with heavy *S. mansoni* infection intensity had the lowest triglyceride levels (*P* = .0004) and lowest diastolic BP (*P* = .01). Participants with moderate *S. mansoni* infection intensity had the lowest LDL-c levels (*P* = .04). Coinfection with multiple (increasing) helminth species was associated with lower total and LDL-c (trend *P* = .02).

**Table 4.** Associations Between Helminth Infection and Metabolic Outcomes

Helminth and Metabolic Outcome	Mean	Crude Mean Difference (95% CI)^a^	*P* Value	Adjusted Mean Difference (95% CI)^b^	*P* Value
HOMA-IR					
* Schistosoma mansoni*, stool Kato-Katz					
* *Uninfected (n = 1065)	GM, 1.90	…		…	
* *Infected (n = 440)	GM, 1.69	−0.05 (−.12 to .02)	.12	0.05 (−.05 to .15)	.28
* S. mansoni*, stool PCR					
* *Uninfected (n = 793)	GM, 1.62	…		…	
* *Infected (n = 694)	GM, 2.06	−0.10 (−.21 to .01)	.07	−0.01 (−.08 to .06)	.77
* S. mansoni* intensity, stool Kato-Katz					
* *Uninfected (n = 1065)	GM, 1.90	…		…	
* *Light (n = 230)	GM, 2.07	0.04 (−.09 to .17)		0.09 (−.07 to .25)	
* *Moderate (n = 121)	GM, 1.20	−0.20 (−.35 to −.05)		−0.04 (−.19 to .12)	
* *Heavy (n = 89)	GM, 1.61	−0.07 (−.21 to .06)	.07	0.07 (−.11 to .25)	.69
* Trichuris trichiura*, stool Kato-Katz					
* *Uninfected (n = 1383)	GM, 1.82	…		…	
* *Infected (n = 122)	GM, 2.00	0.04 (−.08 to .17)	.50	0.08 (−.05 to .20)	.21
* *Hookworm, stool PCR					
* *Uninfected (n = 1309)	GM, 1.83	…		…	
* *Infected (n = 55)	GM, 1.76	−0.02 (−.17 to .14)	.83	0.11 (−.18 to .40)	.45
* Strongyloides stercoralis* stool PCR					
* *Uninfected (n = 1385)	GM, 1.89	…		…	
* *Infected (n = 101)	GM, 1.25	−0.18 (−.40 to .035)	.10	0.04 (−.16 to .24)	.70
* *Infection with multiple helminth species (*S. mansoni* [PCR], *T. trichiura*, hookworm, and *S. stercoralis*)					
* *Helminth uninfected (n = 655)	GM, 2.12	…		…	
* *Infected with any 1 helminth (n = 611)	GM, 1.58	−0.13 (−.23 to −.03)		−0.04 (−.12 to .04)	
* *Infected with any 2 helminths (n = 155)	GM, 1.90	−0.05 (−.18 to .09)		0.13 (.03–.23)	
* *Infected with any 3 or 4 helminths (n = 17)	GM, 1.11	−0.28 (−.58 to .02)	<.01	−0.22 (−.66 to .23)	.05
* *Infection with multiple helminth species (test for trend)	…	−0.07 (−.12 to −.02)	.01	0.01 (−.04 to .06)	.61
Triglycerides (mmol/L)					
* S. mansoni*, stool Kato-Katz					
* *Uninfected (n = 1065)	GM, 1.02	…		…	
* *Infected (n = 440)	GM, 0.90	−0.05 (−.12 to .01)	.12	−0.05 (−.14 to .03)	.21
* S. mansoni*, stool PCR					
* *Uninfected (n = 793)	GM, 1.00	…		…	
* *Infected (n = 694)	GM, 0.95	−0.02 (−.07 to .02)	.28	−0.04 (−.08 to .01)	.09
* S. mansoni* intensity, stool Kato-Katz					
* *Uninfected (n = 1065)	GM, 1.02	…		…	
* *Light (n = 230)	GM, 0.95	−0.03 (−.15 to .08)		−0.05 (−.18 to .08)	
* *Moderate (n = 121)	GM, 0.98	−0.02 (−.11 to .08)		0.00 (−.09 to .09)	
* *Heavy (n = 89)	GM, 0.72	−0.15 (−.23 to −.08)	<.01	−**0.13 (**−**.20 to 0.07)**	**<.01**
* T. trichiura*, stool Kato-Katz					
* *Uninfected (n = 1383)	GM, 0.99	…		…	
* *Infected (n = 122)	GM, 0.87	−0.06 (−.11 to −.00)	.04	−0.05 (−.13 to .04)	.29
* *Hookworm, stool PCR					
* *Uninfected (n = 1309)	GM, 0.97	…		…	
* *Infected (n = 55)	GM, 0.67	−0.17 (−.30 to −.03)	.02	−0.08 (−.25 to .09)	.32
* S. stercoralis*, stool PCR					
* *Uninfected (n = 1385)	GM, 0.97	…		…	
* *Infected (n = 101)	GM, 0.95	−0.01 (−.09 to .08)	.84	−0.05 (−.15 to .05)	.32
* *Infection with multiple helminth species (*S. mansoni* [PCR], *T. trichiura*, hookworm, and *S. stercoralis*)					
* *Helminth uninfected (n = 655)	GM, 0.99	…		…	
* *Infected with any 1 helminth (n = 611)	GM, 0.97	−0.01 (−.04 to .03)		−0.01 (−.05 to .02)	
* *Infected with any 2 helminths (n = 155)	GM, 0.87	−0.05 (−.12 to .01)		−0.07 (−.15 to .02)	
* *Infected with any 3 or 4 helminths (n = 17)	GM, 0.65	−0.18 (−.37 to .01)	.23	−0.20 (−.49 to .09)	.50
* *Infection with multiple helminth species (test for trend)	…	−0.03 (−.05 to .00)	.06	−0.03 (−.07 to .01)	.12
Total cholesterol (mmol/L)					
* S. mansoni*, stool Kato-Katz					
* *Uninfected (n = 1065)	4.64	…		…	
* *Infected (n = 440)	4.24	−0.40 (−.62 to −.19)	<.01	−**0.25 (**−**.44 to** −**.07)**	**.01**
* S. mansoni*, *s*tool PCR					
* *Uninfected (n = 793)	4.69	…		…	
* *Infected (n = 694)	4.32	−0.37 (−.63 to −.11)	.007	−**0.28 (**−**.53 to** −**.03)**	**.03**
* S. mansoni* intensity, stool Kato-Katz					
* *Uninfected (n = 1065)	4.64	…		…	
* *Light (n = 230)	4.34	−0.30 (−.54 to −.07)		−0.19 (−.42 to .05)	
* *Moderate (n = 121)	4.17	−0.48 (−.77 to −.19)		−0.33 (−.59 to −.08)	
* *Heavy (n = 89)	4.10	−0.55 (−.87 to −.23)	.01	−0.33 (−.62 to −.04)	.06
* T. trichiura*, stool Kato-Katz					
* *Uninfected (n = 1383)	4.53	…		…	
* *Infected (n = 122)	4.39	−0.14 (−.42 to .15)	.33	0.00 (−.25 to .24)	.99
* *Hookworm, stool PCR					
* *Uninfected (n = 1309)	4.53	…		…	
* *Infected (n = 55)	4.06	−0.47 (−.76 to −.18)	.003	−0.22 (−.53 to .09)	.16
* S. stercoralis*, stool PCR					
* *Uninfected (n = 1385)	4.53	…		…	
* *Infected (n = 101)	4.24	−0.29 (−.61 to .03)	.07	−0.22 (−.53 to .08)	.15
* *Infection with multiple helminth species (*S. mansoni* [PCR], *T. trichiura*, hookworm, and *S. stercoralis*)					
* *Helminth uninfected (n = 655)	4.71	…		…	
* *Infected with any 1 helminth (n = 611)	4.37	−0.34 (−.62 to −.06)		−**0.27 (**−**.53 to** −**.01)**	
* *Infected with any 2 helminths (n = 155)	4.23	−0.48 (−.83 to −.13)		−**0.32 (**−**.63 to** −**.01)**	
* *Infected with any 3 or 4 helminths (n = 17)	3.75	−0.97 (−1.49 to −.44)	.01	−0.53 (−1.08 to .02)	.11
* *Infection with multiple helminth species (test for trend)	…	−0.29 (−.48 to −.09)	<.01	−**0.20 (**−**.36 to** −**.03)**	**.02**
LDL-c (mmol/L)					
* S. mansoni*, stool Kato-Katz					
* *Uninfected (n = 1065)	2.80	…		…	
* *Infected (n = 440)	2.37	−0.43 (−.74 to −.12)	.01	−**0.25 (**−**.49 to** −**.02)**	**.04**
* S. mansoni*, *s*tool PCR					
* *Uninfected (n = 793)	2.87	…		…	
* *Infected (n = 694)	2.45	−0.42 (−.78 to −.05)	.03	−0.28 (−.57 to .01)	.06
* S. mansoni* intensity, stool Kato-Katz					
* *Uninfected (n = 1065)	2.80	…		…	
* *Light (n = 230)	2.49	−0.32 (−.60 to −.03)		−0.23 (−.46 to .00)	
* *Moderate (n = 121)	2.18	−0.62 (−.97 to −.28)		−**0.39 (**−**.72 to** −**.05)**	
* *Heavy (n = 89)	2.34	−0.46 (−.91 to −.01)	<.01	−0.14 (−.51 to .23)	**.04**
* T. trichiura*, stool Kato-Katz					
* *Uninfected (n = 1383)	2.66	…		…	
* *Infected (n = 122)	2.69	0.03 (−.32 to .37)	.87	0.05 (−.22 to .33)	.7
* *Hookworm, stool PCR					
* *Uninfected (n = 1309)	2.67	…		…	
* *Infected (n = 55)	2.13	−0.55 (−.79 to −.30)	<.01	0.08 (−.19 to .35)	.55
* S. stercoralis*, stool PCR					
* *Uninfected (n = 1385)	2.69	…		…	
* *Infected (n = 101)	2.34	−0.35 (−.58 to −.12)	.004	−**0.32 (**−**.53 to** −**.12)**	**.003**
* *Infection with multiple helminth species (*S. mansoni* [PCR], *T. trichiura*, hookworm, and *S. stercoralis*)					
* *Helminth uninfected (n = 655)	2.90	…		…	
* *Infected with any 1 helminth (n = 611)	2.48	−0.42 (−.82 to −.02)		−0.27 (−.55 to .02)	
* *Infected with any 2 helminths (n = 155)	2.44	−0.46 (−.78 to −.13)		−**0.29 (**−**.54 to** −**.05)**	
* *Infected with any 3 or 4 helminths (n = 17)	1.96	−0.94 (−1.57 to −.31)	.02	−0.52 (−1.14 to .10)	.10
* *Infection with multiple helminth species (test for trend)	…	−0.30 (−.53 to −.07)	.01	−**0.19 (**−**.35 to** −**.04)**	**.02**
HDL-c (mmol/L)					
* S. mansoni*, stool Kato-Katz					
* *Uninfected (n = 1065)	GM, 1.81	…		…	
* *Infected (n = 440)	GM, 1.82	0.09 (−.14 to .14)	.98	0.05 (−.04 to .14)	.26
* S. mansoni*, *s*tool PCR					
* *Uninfected (n = 793)	GM, 1.83	…		…	
* *Infected (n = 694)	GM, 1.79	−0.01 (−.16 to .14)	.92	0.00 (−.09 to .09)	.96
* S. mansoni* intensity, stool Kato-Katz					
* *Uninfected (n = 1065)	GM, 1.81	…		…	
* *Light (n = 230)	GM, 1.80	−0.01 (−.10 to .09)		0.06 (−.03 to .14)	
* *Moderate (n = 121)	GM, 2.19	0.08 (−.08 to .24)		0.12 (−.01 to .26)	
* *Heavy (n = 89)	GM, 1.49	−0.08 (−.34 to .17)	.02	−0.07 (−.22 to .09)	.07
* T. trichiura*, stool Kato-Katz					
* *Uninfected (n = 1383)	GM, 1.83	…		…	
* *Infected (n = 122)	GM, 1.62	−0.06 (−.18 to .07)	.38	0.00 (−.12 to .12)	.97
* *Hookworm, stool PCR					
* *Uninfected (n = 1309)	GM, 1.84	…		…	
* *Infected (n = 55)	GM, 2.02	0.04 (−.18 to .26)	.70	−0.03 (−.13 to .08)	.57
*S. stercoralis*, stool PCR					
* *Uninfected (n = 1385)	GM, 1.81	…		…	
* *Infected (n = 101)	GM, 1.79	−0.01 (−.10 to .09)	.92	−0.04 (−.13 to .05)	.36
* *Infection with multiple helminth species (*S. mansoni* [PCR], *T. trichiura*, hookworm, and *S. stercoralis*)					
* *Helminth uninfected (n = 655)	GM, 1.82	…		…	
* *Infected with any 1 helminth (n = 611)	GM, 1.83	0.00 (−.16 to .17)		0.00 (−.10 to .10)	
* *Infected with any 2 helminths (n = 155)	GM, 1.70	−0.03 (−.16 to .11)		−0.02 (−.11 to .07)	
* *Infected with any 3 or 4 helminths (n = 17)	GM, 1.75	−0.02 (−.24 to .21)	.86	0.05 (−.19 to .28)	.89
* *Infection with multiple helminth species (test for trend)	…	−0.01 (−.10 to .09)	.88	−0.00 (−.06 to .05)	.89
Diastolic BP (mm Hg)					
* S. mansoni*, stool Kato-Katz					
* *Uninfected (n = 1065)	76.30	…		…	
* *Infected (n = 440)	75.86	−0.44 (−1.89 to 1.01)	.54	0.72 (−1.40 to 2.84)	.49
* S. mansoni*, *s*tool PCR					
* *Uninfected (n = 793)	76.35	…		…	
* *Infected (n = 694)	75.88	−0.46 (−1.48 to .56)	.36	0.56 (−.84 to 1.95)	.42
* S. mansoni* intensity, stool Kato-Katz					
* *Uninfected (n = 1065)	76.30	…		…	
* *Light (n = 230)	76.58	0.27 (−1.08 to 1.63)		1.25 (−.71 to 3.22)	
* *Moderate (n = 121)	76.75	0.45 (−2.56 to 3.47)		1.55 (−2.35 to 5.46)	
* *Heavy (n = 89)	72.84	−3.46 (−4.81 to −2.12)	<.01	−**2.29 (**−**3.91 to** −**.68)**	**.01**
* T. trichiura*, stool Kato-Katz					
* *Uninfected (n = 1383)	76.40	…		…	
* *Infected (n = 122)	72.97	−3.43 (−5.64 to −1.22)	<.01	−1.45 (−3.58 to .69)	.18
* *Hookworm, stool PCR					
* *Uninfected (n = 1309)	76.35	…		…	
* *Infected (n = 55)	72.44	−3.90 (−7.22 to −.59)	.02	2.20 (−.92 to 5.33)	.16
* S. stercoralis*, stool PCR					
* *Uninfected (n = 1385)	76.09	…		…	
* *Infected (n = 101)	76.60	0.51 (−2.67 to 3.69)	.74	−0.56 (−3.25 to 2.14)	.68
* *Infection with multiple helminth species (*S. mansoni* [PCR], *T. trichiura*, hookworm, and *S. stercoralis*)					
* *Helminth uninfected (n = 655)	76.44	…		…	
* *Infected with any 1 helminth (n = 611)	76.34	−0.10 (−1.22 to 1.02)		0.87 (−.80 to 2.53)	
* *Infected with any 2 helminths (n = 155)	74.07	−2.37 (−4.33 to −.41)		0.08 (−2.21 to 2.38)	
* *Infected with any 3 or 4 helminths (n = 17)	70.78	−5.66 (−12.24 to .91)	.10	−4.11 (−10.33 to 2.11)	.47
* *Infection with multiple helminth species (test for trend)	…	−0.94 (−1.70 to −.18)	.02	−0.26 (−1.13 to .61)	.54

Abbreviations: BP, blood pressure; CI, confidence interval; GM, geometric mean; HDL-c, high-density lipoprotein cholesterol; HOMA-IR, homeostatic model assessment of insulin resistance; LDL-c, low-density lipoprotein cholesterol; PCR, polymerase chain reaction.

^a^Both crude and adjusted results allowed for the survey design—that is, weighting and clustering.

^b^Adjusted for the following variables: HOMA-IR (age, sex, occupation, residence, lake contact, treatment for worms, treatment with coartem, body mass index [BMI], family history of diabetes mellitus, maternal and paternal tribe); triglycerides (age, sex, occupation, residence, diet, treatment for worms, family history of obesity, paternal and maternal tribe); total cholesterol (age, sex, occupation, residence, diet, exercise, family history of obesity, paternal and maternal tribe); LDL-c (age, sex, occupation, diet, exercise, treatment for worms, treatment with coartem, parental tribe); HDL-c (age, sex, occupation, residence, diet, treatment for worms, treatment with coartem, parental tribe); diastolic BP (age, sex, occupation, residence, lake contact, treatment for worms, paternal tribe, BMI). There was no evidence of association between helminth infection and fasting blood glucose, glycated hemoglobin, systolic BP, BMI, waist circumference, or waist-to-hip ratio (see Supplementary Table 2).

Four serious adverse events (SAEs) were reported in the first 3 years of the anthelminthic intervention [28]. In the fourth year of the intervention, no SAEs were reported.

## DISCUSSION

This is the first trial to investigate effects of an anthelminthic intervention on metabolic outcomes in an *S. mansoni*–endemic, high-transmission setting. We found no effect of 4 years of intensive vs standard anthelminthic treatment on insulin resistance, but there was a modest increase in LDL-c in the intensive vs standard arm. Consistent with this, there was strong observational evidence of association between *S. mansoni* infection and lower levels of total cholesterol and LDL-c. The lowest levels of triglycerides and diastolic BP were observed among participants with heavy *S. mansoni* infection and the lowest levels of LDL-c among those with moderate *S. mansoni* intensity. *Strongyloides* infection was also inversely associated with LDL-c. A dose-response relationship was observed between increasing numbers of helminth species detected and lower cholesterol levels.

The effect of the intervention on LDL-c and consistency of this with our cross-sectional observations strongly suggests a causal link between helminth infection and lipid profiles. Our results, in a setting dominated by schistosomiasis, accord with findings in experimental animals [14, 29] and with associations observed between intestinal nematode infection and lipids in humans [30, 31]. One possible mechanism is direct consumption of lipids by helminths. Schistosomes do not synthesize cholesterol and rely on the host for their supply [32]. However, the effect of schistosomes is likely not limited to direct consumption, with immunological effects implicated. La Flamme et al showed that chronic exposure to *S. mansoni* eggs reduced serum cholesterol in mice with changes due to egg-induced increased uptake of LDL by macrophages [29]. It has been suggested that helminths might modulate production of naturally occurring antibodies to cholesterol [33]. Helminths resident in the lumen might hinder lipid absorption from the gut directly. In addition, there is evidence that helminth infection influences the intestinal microbiome [34], which also impacts blood lipids [35]. Studies investigating the influence of helminths on the microbiome and how that relates to lipid metabolism would provide a deeper understanding of the underlying mechanisms.

An unanticipated finding was the association between heavy *S. mansoni* infection intensity and lower diastolic BP. Essential hypertension accounts for 90% of hypertension cases. The etiology is not well understood, but it is hypothesized that inflammation may cause essential hypertension [36]. Two pathways are proposed. First, inflammation of the renal interstitium results in monocyte/macrophage infiltration of renal perivascular fat, oxidative stress, and loss of peritubular capillaries. This may lead to medullary hypoxia, impaired sodium excretion, and consequently, hypertension [37]. Second, inflammation results in monocyte/macrophage infiltration of arterial walls leading to arterial stiffness. Arterial stiffness triggers angiotensin II, which results in hypertension. Helminths may influence these pathways via their immunomodulatory properties. Helminths may also indirectly influence blood pressure through effects on the intestinal microbiome. Gut microbiota digest fiber and release short-chain fatty acids which, on absorption into the circulation stimulate the release of renin, a major component of the renin angiotensin aldosterone system [38]. We recommend studies to further explore the relationship between helminth infection and BP.

We were surprised to find no impact of anthelminthic treatment or of helminths on insulin resistance, given the strong evidence of effects from animal models and evidence of increased insulin resistance following STH treatment among participants in the Indonesian trial. Perhaps the most probable explanation lies in the transmission dynamics of schistosomiasis in this transmission “hot-spot” setting; and in the biology of schistosomes, as distinct from STH. In this trial, we observed a reduction in helminth prevalence in the standard arm over time, as well as in the intensive arm [25, 28], and this may have weakened any chance of seeing a differential effect of trial interventions on the metabolic outcomes. As well, single stool Kato-Katz analysis has low sensitivity in detecting *S. mansoni* infection. As previously reported, the more sensitive urine circulating cathodic antigen used after 3 years of intervention suggested persistent (albeit reduced intensity) infection in >80% of participants in both trial arms, presumably due to ongoing transmission and reinfection. Moreover, schistosomes differ from STH in that they reside in the vasculature and treatment does not result in immediate expulsion from the body. Immunological changes induced by helminths persist even after treatment of the worms. *Schistosoma* egg antigen elicits reduced insulin resistance in an animal model in the absence of live worms, and *Schistosoma* egg antigen is persistent—liver egg counts were almost unchanged 26 weeks after treatment in a mouse model [39]; in humans, antibody to egg antigen was detectable 8 years after curative treatment of schistosome infection [40]. Therefore, effects of schistosome infection may persist after treatment of adult worms with praziquantel. Furthermore, animal models investigating effects of helminths on insulin resistance have tended to use obese models. By contrast, fishing community populations are generally young, slim, and active and therefore at low risk of metabolic disorders.

Our results in this setting of high schistosomiasis transmission, namely strong observational associations and supportive suggestion of a treatment effect for lipid profiles, contrasting with lack of association or treatment effect for insulin resistance, could be explained if the former depends predominantly upon active schistosome infection and the latter upon exposure to schistosome antigen.

Strengths of our study include the robust trial design, large sample size, and intense helminth exposure. However, ongoing helminth transmission and cluster-level randomization limited our ability to determine effects of helminth elimination and more focused, individually randomized trials would be useful to assess this further. While multiple outcomes were assessed, risking false-positive findings, the coherence of results on lipid profiles within the study, and when compared to other studies, is persuasive. Finally, we cannot rule out a direct effect of praziquantel and albendazole on metabolic outcomes, although no prior evidence suggests this.

Taken together, our findings have important implications. They provide an understanding on how anthelminthic treatment may impact metabolic health, thus increasing the burden of noncommunicable diseases and accelerating the epidemiological transition. Helminth infections may be beneficial to lipid metabolism and BP control in humans. Identification of potentially protective interactions and their underlying mechanisms is important for the development of future therapies.

## Supplementary Data

Supplementary materials are available at *Clinical Infectious Diseases* online. Consisting of data provided by the authors to benefit the reader, the posted materials are not copyedited and are the sole responsibility of the authors, so questions or comments should be addressed to the corresponding author.

ciz859_suppl_Supplementary_Material

ciz859_suppl_Supplementary_Tables

## Notes

***Acknowledgments.*** The authors thank the Koome subcounty community members, their local council leaders and beach management committee members, and village health team members for participating in this study; the Koome Health Centre III for support; and the leadership of Mukono District, particularly the district health officer (Elly Tumushabe) and the councillor for Koome subcounty (Asuman Muwumuza), who are members of the Trial Steering Committee (TSC), for their support. The authors also thank the other members of the TSC: Heiner Grosskurth (chair), Edridah Tukahebwa, Narcis Kabatereine, Neil Pearce, Anatoli Kamali, and Monica Kuteesa; the Calvary Chapel Island Mission for providing voluntary counseling and human immunodeficiency virus (HIV) testing for community members, including study participants, in collaboration with our research program; and Moffat Nyirenda, Stephen Cose, and Ponsiano Ocama, who are members of R. E. S.’s doctoral committee, for their guidance and support. The Lake Victoria Island Intervention Study on Worms and Allergy-Related Diseases (LaVIISWA) trial team. Project leaders and physicians: Richard Sanya, Margaret Nampijja, Harriet Mpairwe, Barbara Nerima; statisticians and data managers: Emily Webb, Lawrence Lubyayi, Hellen Akurut, Justin Okello, Sebastian Owilla, Jacob Ochola; clinical officers: Christopher Zziwa, Milly Namutebi; nurses: Esther Nakazibwe, Josephine Tumusiime, Caroline Ninsiima, Susan Amongi, Grace Kamukama, Susan Iwala, Rita Asherwin, Rehema Nampijja, Florence Akello; internal monitor: Mirriam Akello; field workers: Robert Kizindo, Moses Sewankambo, Denis Nsubuga; laboratory staff and collaborators: Stephen Cose, Prossy Kabuubi Nakawungu, Emmanuel Niwagaba, Gloria Oduru, Grace Kabami, Elson Abayo, Fred Muwonge Kakooza, Joyce Kabagenyi, Gyaviira Nkurunungi, Angela Nalwoga, John Vianney Tushabe, Jacent Nassuuna, Bridgious Walusimbi; Boatman: David Abiriga; Driver: Richard Walusimbi; HIV counseling and testing: Cynthia Kabonesa; vector control program staff: James Kaweesa, Edridah Tukahebwa; administrative management: Moses Kizza; principal investigator: Alison Elliott.

***Disclaimer.*** The funders were not involved in the design of the study, collection, analysis or interpretation of data, writing of the manuscript, or decision to submit it for publication.

***Financial support.*** This work was supported by the Wellcome Trust (grant number 095778) and the Royal Society (grant number IC160132). R. E. S. is supported by a PhD fellowship awarded under the DELTAS Africa Initiative (grant number 107743); the Developing Excellence in Leadership, Training and Science Africa Initiative is an independent funding scheme of the African Academy of Sciences, Alliance for Accelerating Excellence in Science in Africa, and supported by the New Partnership for Africa’s Development Planning and Coordinating Agency with funding from the Wellcome Trust (grant number 107743) and the UK government. The work was conducted at the Medical Research Council (MRC)/Uganda Virus Research Institute and London School of Hygiene and Tropical Medicine Uganda Research Unit, which is jointly funded by the UK MRC and the UK Department for International Development (DFID) under the MRC/DFID Concordat agreement and is also part of the EDCTP2 program supported by the European Union.

***Potential conflicts of interest.*** The authors report no potential conflicts of interest. All authors have submitted the ICMJE Form for Disclosure of Potential Conflicts of Interest. Conflicts that the editors consider relevant to the content of the manuscript have been disclosed.
