# Supplementary material for: The Effect of Helminth Infections and Their Treatment on Metabolic Outcomes: Results of a Cluster-Randomized Trial
Source: Clin Infect Dis. 2019 Aug 30;71(3):601–13. doi: 10.1093/cid/ciz859 (PMC7384320; doi:10.1093/cid/ciz859)
Supplement: ciz859_suppl_Supplementary_Material [file ciz859_suppl_supplementary_material.docx]

**SUPPLEMENTARY METHODS**

**Sampling procedures for the household survey**

We selected 70 households per village by simple random sampling using STATA software (College Station Texas, US). The study team, with support from the village health team members, assigned numbers to households and maintained registers of household members. The registers were updated regularly throughout the trial period. This numbering system was then used as the sampling frame for the household survey.

**Measurement of weight, height, waist and hip circumference, and blood pressure**

With the participants wearing light clothing, body weight was measured using a portable flat digital scale (SECA model 875 7021094, Hamburg, Germany) and recorded to the nearest 0.1kg. Height was measured using a portable stadiometer (SECA model 213 1721009, Hamburg, Germany) and recorded to the nearest 0.1cm. Waist circumference (measured at the mid-way position between the iliac crest and lowest rib) and hip circumference (measured at the level of the greater trochanters) were measured using a non-stretchable tape and recorded to the nearest 0.1cm. The stadiometers and weighing scales were checked daily against standard calibration rods and weights respectively.

Blood pressure was measured using a digital sphygmomanometer (OMRON Model M2[hem-7121-E], Omron Health Care, Kyoto, Japan) of appropriate cuff size. Each participant sat comfortably for five minutes, after which three readings of systolic and diastolic pressure were taken five minutes apart. The average of the second and third reading was used in the analysis. For quality control, the blood pressure machines were calibrated by the Uganda National Bureau of Standards.

**Measurement of fasting plasma glucose, insulin, fasting lipid profile, glycated haemoglobin (HbA1c)**

Peripheral venous blood samples were collected after an overnight fast in EDTA, sodium fluoride and serum-separator tubes. Participants were requested to fast overnight for at least eight hours and not to exercise or smoke before sample collection in the morning. An aliquot of whole blood from the EDTA tube was obtained and kept at room temperature for the measurement of HbA1c. Aliquots of plasma from the blood samples in the sodium fluoride and EDTA tubes and serum from samples in the serum-separator tubes were collected within an hour after phlebotomy and immediately stored in liquid nitrogen. The other tests were carried out at the MRC/UVRI and LSHTM Uganda Research Unit Clinical and Diagnostic Laboratory in Entebbe. The *cobas* 6000 analyser (*cobas* c 501 module, Roche Diagnostics, Rotkreuz, Switzerland) was used to perform the tests. Fasting plasma glucose was measured using the enzymatic method. Serum lipid levels (total serum cholesterol, triglyceride levels, HDL-cholesterol and LDL-cholesterol) were measured using the enzymatic colorimetric method. Glycated haemoglobin was measured using the turbidimetric inhibition immunoassay for haemolysed whole blood.

**Stool analysis**

The Kato Katz result was used as the main assessment of *S. mansoni* and *T. trichiura* infection status [1]. Stool real-time polymerase chain reaction (PCR) was also used to detect *S. mansoni*, hookworm (*Necator Americanus*) and *Strongyloides Stercoralis* infection [2, 3]. Due to logistical issues, the time between sample preparation and reading of the Kato Katz slides was variable and therefore only hookworm PCR results are presented; *S. stercoralis* cannot be detected by Kato Katz so was assessed only by PCR.

**Sample size**

For this survey, we planned to recruit 1950 participants (half in each study arm; 75 participants aged ≥10 years per village). This sample size would have 80% power to detect a difference of 0.05 in mean HOMA-IR (similar to that detected between individuals with and without helminth infection in a cross-sectional study in Indonesia [4]) assuming an intra-cluster correlation coefficient (ICC) of 0.03 and a standard deviation on the log scale of 0.2. With a higher ICC of 0.1, we would have 80% power to detect a difference of 0.075 in mean HOMA-IR between the two trial arms.

**Further details of confounders considered for observational analyses**

The individual level observational analysis was performed to explore helminths as risk factors for metabolic disease. Helminths were considered as the main exposures of interest. Potential confounders were identified based on a causal diagram (supplementary figure 1).

**Supplementary figure 1: Causal diagram showing risk factors for helminths and metabolic outcomes**

All outcomes were adjusted for age, sex, residence (having lived in the study area for the last four years), frequency of contact with the lake, prior treatment for worms and treatment with *Coartem* (artemether/lumefantrine). In addition, potential confounders considered for HOMA-IR, HbA1c and glucose were family history of diabetes, paternal and maternal tribe, occupation and exercise. For lipids, BMI, waist circumference and waist-hip ratio, additional potential confounders were occupation, diet, exercise, family history of obesity, paternal and maternal tribe. For blood pressure additional potential confounders were BMI, occupation, exercise, family history of hypertension, paternal and maternal tribe. Associations were estimated using linear regression models fitted for each worm separately. For *S. mansoni* intensity, differences between categories of intensity (mild, moderate and heavy) and the uninfected were analysed without assuming a trend. Stata “svy” commands were used to allow for the clustering of participants within villages, and for the non-self-weighting survey design due to variable village sizes. Univariate and multivariable analyses were performed. Crude and adjusted coefficients were obtained using linear regression. For the adjusted analysis, only risk factors/ confounders associated with the outcome with p-value≤0.15 from the crude analysis were included in the final models. No adjustments were performed for multiple testing.

**CONSORT 2010 checklist of information to include when reporting a cluster randomised trial**

| Section/Topic | Item No | Standard Checklist item | Extension for cluster designs | Page No * |
| --- | --- | --- | --- | --- |
| Title and abstract | | | |  |
|  | 1a | Identification as a randomised trial in the title | Identification as a cluster randomised trial in the title | Page 1 |
|  | 1b | Structured summary of trial design, methods, results, and conclusions (for specific guidance see CONSORT for abstracts)^[[1]](#endnote-1),^^[[2]](#endnote-2)^ | See table 2 | Page 3 |
| Introduction | | | |  |
| Background and objectives | 2a | Scientific background and explanation of rationale | Rationale for using a cluster design | Page 5-7 |
|  | 2b | Specific objectives or hypotheses | Whether objectives pertain to the cluster level, the individual participant level or both | Page 6-7 |
| Methods | | | |  |
| Trial design | 3a | Description of trial design (such as parallel, factorial) including allocation ratio | Definition of cluster and description of how the design features apply to the clusters | Page 7 |
|  | 3b | Important changes to methods after trial commencement (such as eligibility criteria), with reasons |  | Page 7 |
| Participants | 4a | Eligibility criteria for participants | Eligibility criteria for clusters | Page 7 |
|  | 4b | Settings and locations where the data were collected |  | Page 7 |
| Interventions | 5 | The interventions for each group with sufficient details to allow replication, including how and when they were actually administered | Whether interventions pertain to the cluster level, the individual participant level or both | Page 7 |
| Outcomes | 6a | Completely defined pre-specified primary and secondary outcome measures, including how and when they were assessed | Whether outcome measures pertain to the cluster level, the individual participant level or both | Page 7-8 |
|  | 6b | Any changes to trial outcomes after the trial commenced, with reasons |  | Page 7 |
| Sample size | 7a | How sample size was determined | Method of calculation, number of clusters(s) (and whether equal or unequal cluster sizes are assumed), cluster size, a coefficient of intracluster correlation (ICC or *k*), and an indication of its uncertainty | Page 7 |
|  | 7b | When applicable, explanation of any interim analyses and stopping guidelines |  | N/A |
| Randomisation: | | | |  |
| Sequence generation | 8a | Method used to generate the random allocation sequence |  | Page 5 |
|  | 8b | Type of randomisation; details of any restriction (such as blocking and block size) | Details of stratification or matching if used | Page 5 |
| Allocation concealment mechanism | 9 | Mechanism used to implement the random allocation sequence (such as sequentially numbered containers), describing any steps taken to conceal the sequence until interventions were assigned | Specification that allocation was based on clusters rather than individuals and whether allocation concealment (if any) was at the cluster level, the individual participant level or both | Page 5 |
| Implementation | 10 | Who generated the random allocation sequence, who enrolled participants, and who assigned participants to interventions | Replace by 10a, 10b and 10c |  |
|  | 10a |  | Who generated the random allocation sequence, who enrolled clusters, and who assigned clusters to interventions | Page 7 |
|  | 10b |  | Mechanism by which individual participants were included in clusters for the purposes of the trial (such as complete enumeration, random sampling) | Page 7 |
|  | 10c |  | From whom consent was sought (representatives of the cluster, or individual cluster members, or both), and whether consent was sought before or after randomisation | Page 7-8 |
|  |  |  |  |  |
| Blinding | 11a | If done, who was blinded after assignment to interventions (for example, participants, care providers, those assessing outcomes) and how |  | N/A |
|  | 11b | If relevant, description of the similarity of interventions |  | N/A |
| Statistical methods | 12a | Statistical methods used to compare groups for primary and secondary outcomes | How clustering was taken into account | Pages 9 |
|  | 12b | Methods for additional analyses, such as subgroup analyses and adjusted analyses |  | Pages 9 |
| Results | | | |  |
| Participant flow (a diagram is strongly recommended) | 13a | For each group, the numbers of participants who were randomly assigned, received intended treatment, and were analysed for the primary outcome | For each group, the numbers of clusters that were randomly assigned, received intended treatment, and were analysed for the primary outcome | Figure 1 |
|  | 13b | For each group, losses and exclusions after randomisation, together with reasons | For each group, losses and exclusions for both clusters and individual cluster members | N/A |
| Recruitment | 14a | Dates defining the periods of recruitment and follow-up |  | Page 10 |
|  | 14b | Why the trial ended or was stopped |  | N/A |
| Baseline data | 15 | A table showing baseline demographic and clinical characteristics for each group | Baseline characteristics for the individual and cluster levels as applicable for each group | Table 1 |
| Numbers analysed | 16 | For each group, number of participants (denominator) included in each analysis and whether the analysis was by original assigned groups | For each group, number of clusters included in each analysis | Tables 2, 3 and 4 |
| Outcomes and estimation | 17a | For each primary and secondary outcome, results for each group, and the estimated effect size and its precision (such as 95% confidence interval) | Results at the individual or cluster level as applicable and a coefficient of intracluster correlation (ICC or k) for each primary outcome | Tables 2, 3 and 4 |
|  | 17b | For binary outcomes, presentation of both absolute and relative effect sizes is recommended |  |  |
| Ancillary analyses | 18 | Results of any other analyses performed, including subgroup analyses and adjusted analyses, distinguishing pre-specified from exploratory |  | Supplementary table 1 |
| Harms | 19 | All important harms or unintended effects in each group (for specific guidance see CONSORT for harms^[[3]](#endnote-3)^) |  | Page 11 |
| Discussion | | | |  |
| Limitations | 20 | Trial limitations, addressing sources of potential bias, imprecision, and, if relevant, multiplicity of analyses |  | Page 13-14 |
| Generalisability | 21 | Generalisability (external validity, applicability) of the trial findings | Generalisability to clusters and/or individual participants (as relevant) | Page 12-14 |
| Interpretation | 22 | Interpretation consistent with results, balancing benefits and harms, and considering other relevant evidence |  | Page 12-14 |
| Other information | | |  |  |
| Registration | 23 | Registration number and name of trial registry |  | Pages 4,7 |
| Protocol | 24 | Where the full trial protocol can be accessed, if available |  | Page 7 |
| Funding | 25 | Sources of funding and other support (such as supply of drugs), role of funders |  | Page 15 |

** Note: page numbers optional depending on journal requirements*

**References**

1. Katz N, Chaves A, Pellegrino J. A simple device for quantitative stool thick-smear technique in Schistosomiasis mansoni. Rev Inst Med Trop Sao Paulo **1972**; 14(6): 397-400.

2. Verweij JJ, Brienen EA, Ziem J, Yelifari L, Polderman AM, Van Lieshout L. Simultaneous detection and quantification of Ancylostoma duodenale, Necator americanus, and Oesophagostomum bifurcum in fecal samples using multiplex real-time PCR. The American journal of tropical medicine and hygiene **2007**; 77(4): 685-90.

3. Verweij JJ, Canales M, Polman K, et al. Molecular diagnosis of Strongyloides stercoralis in faecal samples using real-time PCR. Transactions of the Royal Society of Tropical Medicine and Hygiene **2009**; 103(4): 342-6.

4. Wiria AE, Hamid F, Wammes LJ, et al. Infection with Soil-Transmitted Helminths Is Associated with Increased Insulin Sensitivity. PloS one **2015**; 10(6): e0127746.

1. [↑](#endnote-ref-1)
2. [↑](#endnote-ref-2)
3. [↑](#endnote-ref-3)
