# Supplementary material for: The Effect of Helminth Infections and Their Treatment on Metabolic Outcomes: Results of a Cluster-Randomized Trial
Source: Clin Infect Dis. 2019 Aug 30;71(3):601–13. doi: 10.1093/cid/ciz859 (PMC7384320; doi:10.1093/cid/ciz859)
Supplement: ciz859_suppl_Supplementary_Tables [file ciz859_suppl_supplementary_tables.docx]

**Supplementary table 1: Cluster - level analysis of metabolic outcomes (by disease categories)**

| **Outcome** | **%** | | **Crude**  **risk ratio (95% CI)** | **P-value** | **Adjusted**  **risk ratio (95% CI)*** | **P-value*** |
| --- | --- | --- | --- | --- | --- | --- |
|  | **Intensive** | **Standard** |  |  |  |  |
| Diabetes (FBG ≥ 7 mmol/l) | 1.2 | 0.6 | 1.99 (0.63, 6.35) | 0.20 | 1.82 (0.56, 5.89) | 0.26 |
| Impaired fasting glucose (FBG 6.1 - 6.9 mmol/l) | 2.5 | 1.6 | 1.56 (0.67, 3.60) | 0.28 | 1.54 (0.67, 3.58) | 0.29 |
| Hypertension** | 8.1 | 9.1 | 0.89 (0.62, 1.29) | 0.52 | 0.85 (0.59, 1.22) | 0.38 |
| Underweight (BMI <18.5)*** | 7.1 | 10.2 | 0.70 (0.46, 1.05) | 0.09 | 0.82 (0.54, 1.24) | 0.32 |
| Overweight (BMI 25.0 – 29.9)*** | 21.3 | 23.2 | 0.92 (0.74, 1.14) | 0.41 | 0.89 (0.73, 1.09) | 0.24 |
| Obese (BMI ≥ 30)*** | 7.7 | 10.1 | 0.86 (0.58, 1.26) | 0.42 | 0.85 (0.56, 1.30) | 0.44 |
| Normal weight (BMI 18.5 – 24.9)*** | 63.9 | 56.5 | 1 |  | 1 |  |
| Central (abdominal) obesity (WC≥94cm males WC ≥80cm Females)*** | 26.8 | 29.4 | 0.91 (0.78, 1.06) | 0.22 | 0.9 (0.79, 1.02) | 0.10 |
| Central (abdominal) obesity (WHR>0.9 males WHR>0.85 Females)*** | 27.3 | 28.4 | 0.96 (0.82, 1.13) | 0.63 | 0.96 (0.80, 1.14) | 0.61 |
| Metabolic Syndrome**** | 6.7 | 5.8 | 0.86 (0.58, 1.28) | 0.43 | 0.83 (0.55, 1.27) | 0.37 |

*FBG, Fasting Blood Glucose; SBP, Systolic Blood Pressure; DBP, Diastolic Blood Pressure; BMI, Body Mass Index; WC, Waist Circumference; WHR, Waist Hip Ratio. *Adjusted for age and sex; ** SBP ≥ 140mmHg or DBP ≥ 90mmHg for adults; Blood pressure ≥90^th^ percentile for participants aged 10-17 years.*** Participants who reported being pregnant at the time of anthropometry were excluded in this analysis. ****Any three of: Central obesity [WC≥94cm males WC ≥80cm Females], FBG>5.55, [SBP≥130 or DBP≥85], Elevated Triglycerides [≥1.7 mmol/L], Reduced HDL – Cholesterol [<1 mmo/L Males, <1.3 mmol/L females]*

**Supplementary table 2: Associations between helminth infection and all metabolic outcomes**

|  |  | **Mean** | **Crude mean difference (95% CI)*** | **P-value*** | **Adjusted mean difference (95% CI)*** | **P-value**** |
| --- | --- | --- | --- | --- | --- | --- |
| **HOMA-IR** |  |  |  |  |  |  |
| *S. mansoni,* stool KK | Uninfected (n=1065) | GM 1.90 |  |  |  |  |
|  | Infected (n=440) | GM 1.69 | -0.05 (-0.12, 0.02) | 0.12 | 0.05 (-0.05, 0.15) | 0.28 |
| *S. mansoni,* stool PCR | Uninfected (n=793) | GM 1.62 |  |  |  |  |
|  | Infected (n=694) | GM 2.06 | -0.10 (-0.21, 0.01) | 0.07 | -0.01 (-0.08, 0 .06) | 0.77 |
| *S. mansoni* intensity, stool KK | Uninfected (n=1065) | GM 1.90 |  |  |  |  |
|  | Light (n=230) | GM 2.07 | 0.04 (-0.09, 0.17) |  | 0.09 ( -0.07, 0.25) |  |
|  | Moderate (n=121) | GM 1.20 | -0.20 (-0.35, -0.05) |  | -0.04 (-0.19, 0.12) |  |
|  | Heavy (n=89) | GM 1.61 | -0.07 (-0.21, 0.06) | 0.07 | 0.07 (-0.11, 0.25) | 0.69 |
| *T. trichiura,* stool KK | Uninfected (n=1383) | GM 1.82 |  |  |  |  |
|  | Infected (n=122) | GM 2.00 | 0.04 (-0.08 0.17) | 0.5 | .077 (-0.05, 0.20) | 0.21 |
| Hookworm, stool PCR | Uninfected (n=1309) | GM 1.83 |  |  |  |  |
|  | Infected (n=55) | GM 1.76 | -0.02 (-0.17, 0.14) | 0.83 | 0.11, (-0.18, 0 .40) | 0.45 |
| *Strongyloides stercoralis,* stool PCR | Uninfected (n=1385) | GM 1.89 |  |  |  |  |
|  | Infected (n=101) | GM 1.25 | -0.18 (-0.40, 0.035) | 0.10 | 0.04 (-0.16, 0.24) | 0.70 |
| Infection with multiple helminth species (*S. mansoni* [PCR], *T. trichiura*, Hookworm and *S. stercoralis)* | Helminth uninfected (n=655) | GM 2.12 |  |  |  |  |
|  | Infected with any one helminth (n=611) | GM 1.58 | -0.13 (-0.23, -0.03) |  | -0.04 (-0.12, 0.04) |  |
|  | Infected with any two helminths (n=155) | GM 1.90 | -0.05 (-0.18, 0.09) |  | 0.13 (0.03, 0.23) |  |
|  | Infected with any three or four helminths (n=17) | GM 1.11 | -0.28 (-0.58, 0.02) | <0.01 | -0.22 (-0.66, 0.23) | 0.05 |
| Infection with multiple helminth species (testing for trend) |  |  | -0.07 (-0.12, -0.02) | 0.01 | 0.01 (-0.04, 0.06) | 0.61 |
| **Fasting blood glucose (mmol/L)** |  |  |  |  |  |  |
| *S. mansoni,* stool KK | Uninfected (n=1065) | 4.85 |  |  |  |  |
|  | Infected (n=440) | 4.74 | -0.10 (-0.21, 0.01) | 0.07 | -0.01 (-0.17, 0.15) | 0.87 |
| *S. mansoni,* stool PCR | Uninfected (n=793) | 4.88 |  |  |  |  |
|  | Infected (n=694) | 4.74 | -0.15 (-0.30, 0.01) | 0.07 | -0.06 (-0.24, 0.12) | 0.47 |
| *S. mansoni* intensity, stool KK | Uninfected (n=1065) | 4.85 |  |  |  |  |
|  | Light (n=230) | 4.83 | -.018 (-0.13, 0.10) |  | 0.06 (-0.10, 0.22) |  |
|  | Moderate (n=121) | 4.68 | -0.17 (-0.31, -0.02) |  | -0.06 (-0.31, 0.19) |  |
|  | Heavy (n=89) | 4.61 | -0.23 (-0.37, -0.09) | <0.01 | -0.15 (-0.33, 0.03) | 0.15 |
| *T. trichiura,* stool KK | Uninfected (n=1383) | 4.81 |  |  |  |  |
|  | Infected (n=122) | 4.80 | -0.01 (-0.26, 0.23) | 0.91 | 0.07 (-0.24, 0.39) | 0.64 |
| Hookworm, stool PCR | Uninfected (n=1309) | 4.82 |  |  |  |  |
|  | Infected (n=55) | 4.67 | -0.15 (-0.34, 0.04) | 0.12 | 0.01 (-0.35, 0.37) | 0.94 |
| *Strongyloides stercoralis,* stool PCR | Uninfected (n=1385) | 4.81 |  |  |  |  |
|  | Infected (n=101) | 4.84 | 0.03 (-0.18, 0.24) | 0.74 | 0.10 (-0.09, 0.29) | 0.29 |
| Infection with multiple helminth species (*S. mansoni* [PCR], *T. trichiura*, Hookworm and *S. stercoralis)* | Helminth uninfected (n=655) | 4.92 |  |  |  |  |
|  | Infected with any one helminth (n=611) | 4.71 | -0.21 (-0.37, -0.05) |  | -0.13 (-0.30, 0.03) |  |
|  | Infected with any two helminths (n=155) | 4.82 | -0.10 (-0.34, 0.14) |  | 0.05 (-0.22, 0.33) |  |
|  | Infected with any three or four helminths (n=17) | 4.91 | -0.01 (-0.40, 0.37) | 0.03 | 0.17 (-0.39, 0.72) | 0.02 |
| Infection with multiple helminth species (testing for trend) |  |  | -0.09 (-0.19, 0.00) | 0.06 | -0.02 (-0.14, 0.10) | 0.73 |
| **Glycated haemoglobin (mmol/m)** |  |  |  |  |  |  |
| *S. mansoni,* stool KK | Uninfected (n=1065) | 31.09 |  |  |  |  |
|  | Infected (n=440) | 30.76 | -0.34 (-1.76, 1.08) | 0.63 | -0.07 (-1.35, 1.21) | 0.91 |
| *S. mansoni,* stool PCR | Uninfected (n=793) | 31.23 |  |  |  |  |
|  | Infected (n=694) | 30.69 | -0.54 (-1.74, 0.65) | 0.36 | -0.20 (-1.46, 1.07) | 0.75 |
| *S. mansoni* intensity, stool KK | Uninfected (n=1065) | 31.09 |  |  |  |  |
|  | Light (n=230) | 30.56 | -0.53 (-2.23, 1.17) |  | -0.32 (-1.91, 1.28) |  |
|  | Moderate (n=121) | 31.08 | -0.02 (-1.58, 1.55) |  | 0.16 (-1.62, 1.94) |  |
|  | Heavy (n=89) | 30.83 | -0.27 (-1.60, 1.07) | 0.90 | 0.35 (-1.11, 1.81) | 0.89 |
| *T. trichiura,* stool KK | Uninfected (n=1383) | 30.91 |  |  |  |  |
|  | Infected (n=122) | 32.00 | 1.09 (-1.13, 3.30) | 0.32 | 2.12 ( -0.14, 4.37) | 0.06 |
| Hookworm, stool PCR | Uninfected (n=1309) | 30.96 |  |  |  |  |
|  | Infected (n=55) | 32.37 | 1.41 (-1.51, 4.34) | 0.33 | 0.21 (-2.26, 2.69) | 0.86 |
| *Strongyloides stercoralis,* stool PCR | Uninfected (n=1385) | 30.90 |  |  |  |  |
|  | Infected (n=101) | 32.20 | 1.30 (-0.84, 3.44) | 0.22 | 1.08 (-1.36, 3.53) | 0.37 |
| Infection with multiple helminth species (*S. mansoni* [PCR], *T. trichiura*, Hookworm and *S. stercoralis)* | Helminth uninfected (n=655) | 31.10 |  |  |  |  |
|  | Infected with any one helminth (n=611) | 30.71 | -0.39 (-1.43, 0.65) |  | 0.01 (-1.18, 1.19) |  |
|  | Infected with any two helminths (n=155) | 31.64 | 0.54 (-1.68, 2.75) |  | 0.77 (-1.98, 3.53) |  |
|  | Infected with any three or four helminths (n=17) | 32.59 | 1.49 (-2.05, 5.04) | 0.29 | 2.39 (-2.62, 7.39) | 0.68 |
| Infection with multiple helminth species (testing for trend) |  |  | 0.09 (-0.72, 0.91) | 0.82 | 0.32 (-0.68, 1.31) | 0.52 |
| **Triglycerides (mmol/L)** |  |  |  |  |  |  |
| *S. mansoni* Stool KK | Uninfected (n=1065) | GM 1.02 |  |  |  |  |
|  | Infected (n=440) | GM 0.90 | -0.05 (-0.12, 0.01) | 0.12 | -0.05 (-0.14, 0.03) | 0.21 |
| *S. mansoni,* stool PCR | Uninfected (n=793) | GM 1.00 |  |  |  |  |
|  | Infected (n=694) | GM 0.95 | -0.02 (-0.07, 0.02) | 0.28 | -0.04 (-0.08, 0.01) | 0.09 |
| *S. mansoni* intensity, stool KK | Uninfected (n=1065) | GM 1.02 |  |  |  |  |
|  | Light (n=230) | GM 0.95 | -0.03 (-0.15, 0.08) |  | -0.05 (-0.18, 0.08) |  |
|  | Moderate (n=121) | GM 0.98 | -0.02 (-0.11, 0.08) |  | 0.00 (-0.09, 0.09) |  |
|  | Heavy (n=89) | GM 0.72 | -0.15 (-0.23, -0.08) | <0.01 | **-0.13 (-0.20, -0.07)** | <0.01 |
| *T. trichiura,* stool KK | Uninfected (n=1383) | GM 0.99 |  |  |  |  |
|  | Infected (n=122) | GM 0.87 | -0.06 (-0.11, -0.00) | 0.04 | -0.05 ( -0.13, 0.04) | 0.29 |
| Hookworm, stool PCR | Uninfected (n=1309) | GM 0.97 |  |  |  |  |
|  | Infected (n=55) | GM 0.67 | -0.17 (-0.30, -0.03) | 0.02 | -0.08 (-0.25, 0.09) | 0.32 |
| *Strongyloides stercoralis,* stool PCR | Uninfected (n=1385) | GM 0.97 |  |  |  |  |
|  | Infected (n=101) | GM 0.95 | -0.01 (-0.09, 0.08) | 0.84 | -0.05 (-0.15, 0.05) | 0.32 |
| Infection with multiple helminth species (*S. mansoni* [PCR], *T. trichiura*, Hookworm and *S. stercoralis)* | Helminth uninfected (n=655) | GM 0.99 |  |  |  |  |
|  | Infected with any one helminth (n=611) | GM 0.97 | -0.01 (-0.04, 0.03) |  | -0.01 (-0.05, 0.02) |  |
|  | Infected with any two helminths (n=155) | GM 0.87 | -0.05 (-0.12, 0.01) |  | -0.07 (-0.15, 0.02) |  |
|  | Infected with any three or four helminths (n=17) | GM 0.65 | -0.18 (-0.37, 0.01) | 0.23 | -0.20 (-0.49, 0.09) | 0.50 |
| Infection with multiple helminth species (testing for trend) |  |  | -0.03 (-0.05, 0.00) | 0.06 | -0.03 (-0.07, 0.01) | 0.12 |
| **Total cholesterol (mmol/L)** |  |  |  |  |  |  |
| *S. mansoni,* stool KK | Uninfected (n=1065) | 4.64 |  |  |  |  |
|  | Infected (n=440) | 4.24 | -0.40 (-0.62, -0.19) | <0.01 | **-0.25 (-0.44, -0.07)** | **0.01** |
| *S. mansoni,* stool PCR | Uninfected (n=793) | 4.69 |  |  |  |  |
|  | Infected (n=694) | 4.32 | -0.37 (-0.63, -0.11) | <0.01 | **-0.28 (-0.53, -0.03)** | **0.03** |
| *S. mansoni* intensity, stool KK | Uninfected (n=1065) | 4.64 |  |  |  |  |
|  | Light (n=230) | 4.34 | -0.30 (-0.54, -0.07) |  | -0.19 (-0.42, 0.05) |  |
|  | Moderate (n=121) | 4.17 | -0.48 ( -0.77, -0.19) |  | -0.33 (-0.59, -0.08) |  |
|  | Heavy (n=89) | 4.10 | -0.55 (-0.87, -0.23) | 0.01 | -0.33 (-0.62, -0.04) | 0.06 |
| *T. trichiura,* stool KK | Uninfected (n=1383) | 4.53 |  |  |  |  |
|  | Infected (n=122) | 4.39 | -0.14 (-0.42, 0.15) | 0.33 | -0.00 (-0.25, 0.24) | 0.99 |
| Hookworm, stool PCR | Uninfected (n=1309) | 4.53 |  |  |  |  |
|  | Infected (n=55) | 4.06 | -0.47 (-0.76, -0.18) | <0.01 | -0.22 (-0.53, 0.09) | 0.16 |
| *Strongyloides stercoralis,* stool PCR | Uninfected (n=1385) | 4.53 |  |  |  |  |
|  | Infected (n=101) | 4.24 | -0.29 (-0.61, 0.03) | 0.07 | -0.22 (-0.53, 0.08) | 0.15 |
| Infection with multiple helminth species (*S. mansoni* [PCR], *T. trichiura*, Hookworm and *S. stercoralis)* | Helminth uninfected (n=655) | 4.71 |  |  |  |  |
|  | Infected with any one helminth (n=611) | 4.37 | -0.34 (-0.62, -0.06) |  | **-0.27 (-0.53, -0.01)** |  |
|  | Infected with any two helminths (n=155) | 4.23 | -0.48 (-0.83, -0.13) |  | **-0.32 (-0.63, -0.01)** |  |
|  | Infected with any three or four helminths (n=17) | 3.75 | -0.97 (-1.49, -0.44) | 0.01 | -0.53 (-1.08, 0.02) | 0.11 |
| Infection with multiple helminth species (testing for trend) |  |  | -0.29 (-0.48, -0.09) | <0.01 | **-0.20 (-0.36, -0.03)** | **0.02** |
| **LDL – Cholesterol (mmol/L)** |  |  |  |  |  |  |
| *S. mansoni,* stool KK | Uninfected (n=1065) | 2.80 |  |  |  |  |
|  | Infected (n=440) | 2.37 | -0.43 (-0.74, -0.12) | 0.01 | **-0.25 ( -0.49, -0.02)** | **0.04** |
| *S. mansoni,* stool PCR | Uninfected (n=793) | 2.87 |  |  |  |  |
|  | Infected (n=694) | 2.45 | -0.42 (-0.78, -0.05) | 0.03 | -0.28 (-0.57, 0.01) | 0.06 |
| *S. mansoni* intensity, stool KK | Uninfected (n=1065) | 2.80 |  |  |  |  |
|  | Light (n=230) | 2.49 | -0.32 (-0.60, -0.03) |  | -0.23 (-0.46, 0.00) |  |
|  | Moderate (n=121) | 2.18 | -0.62 (-0.97, -0.28) |  | -0.39 (-0.72, -0.05) |  |
|  | Heavy (n=89) | 2.34 | -0.46 (-0.91, -0.01) | 0.001 | -0.14 ( -0.51, 0.23) | **0.04** |
| *T. trichiura,* stool KK | Uninfected (n=1383) | 2.66 |  |  |  |  |
|  | Infected (n=122) | 2.69 | 0.03 (-0.32, 0.37) | 0.87 | 0.05 (-0.22, 0.33) | 0.70 |
| Hookworm, stool PCR | Uninfected (n=1309) | 2.67 |  |  |  |  |
|  | Infected (n=55) | 2.13 | -0.55 (-0.79, -0.30) | <0.01 | 0.08 (-0.19, 0.35) | 0.55 |
| *Strongyloides stercoralis,* stool PCR | Uninfected (n=1385) | 2.69 |  |  |  |  |
|  | Infected (n=101) | 2.34 | -0.35 (-0.58, -0.12) | <0.01 | **-0.32 (-0.53, -0.12)** | **<0.01** |
| Infection with multiple helminth species (*S. mansoni* [PCR], *T. trichiura*, Hookworm and *S. stercoralis)* | Helminth uninfected (n=655) | 2.90 |  |  |  |  |
|  | Infected with any one helminth (n=611) | 2.48 | -0.42 (-0.82, -0.02) |  | -0.27 (-0.55, 0.02) |  |
|  | Infected with any two helminths (n=155) | 2.44 | -0.46 (-0.78, -0.13) |  | **-0.29 (-0.54, -0.05)** |  |
|  | Infected with any three or four helminths (n=17) | 1.96 | -0.94 (-1.57, -0.31) | 0.02 | -0.52 (-1.14, 0.10) | 0.10 |
| Infection with multiple helminth species (testing for trend) |  |  | -0.30 (-0.53, -0.07) | 0.01 | **-0.19 (-0.35, -0.04)** | **0.02** |
| **HDL – Cholesterol (mmol/L)** |  |  |  |  |  |  |
| *S. mansoni,* stool KK | Uninfected (n=1065) | GM 1.81 |  |  |  |  |
|  | Infected (n=440) | GM 1.82 | 0.09 (-0.14, 0.14) | 0.98 | 0.05 (-0.04, 0.14) | 0.26 |
| *S. mansoni,* stool PCR | Uninfected (n=793) | GM 1.83 |  |  |  |  |
|  | Infected (n=694) | GM 1.79 | -0.01 (-0.16, 0.14) | 0.92 | 0.00 (-0.09, 0.09) | 0.96 |
| *S. mansoni* intensity, stool KK | Uninfected (n=1065) | GM 1.81 |  |  |  |  |
|  | Light (n=230) | GM 1.80 | -0.01 (-0.10, 0.09) |  | 0.06 (-0.03, 0.14) |  |
|  | Moderate (n=121) | GM 2.19 | 0.08 (-0.08, 0.24) |  | 0.12 (-0.01, 0.26) |  |
|  | Heavy (n=89) | GM 1.49 | -0.08 (-0.34, 0.17) | 0.02 | -0.07 (-0.22, 0.09) | 0.07 |
| *T. trichiura,* stool KK | Uninfected (n=1383) | GM 1.83 |  |  |  |  |
|  | Infected (n=122) | GM 1.62 | -0.06 (-0.18, 0.07) | 0.38 | 0.00 (-0.12, 0.12) | 0.97 |
| Hookworm, stool PCR | Uninfected (n=1309) | GM 1.84 |  |  |  |  |
|  | Infected (n=55) | GM 2.02 | 0.04 (-0.18, 0.26) | 0.70 | -0.03 (-0.13, 0.08) | 0.57 |
| *Strongyloides stercoralis,* stool PCR | Uninfected (n=1385) | GM 1.81 |  |  |  |  |
|  | Infected (n=101) | GM 1.79 | -0.01 (-0.10, 0.09) | 0.92 | -0.04 (-0.13, 0.05) | 0.36 |
| Infection with multiple helminth species (*S. mansoni* [PCR], *T. trichiura*, Hookworm and *S. stercoralis)* | Helminth uninfected (n=655) | GM 1.82 |  |  |  |  |
|  | Infected with any one helminth (n=611) | GM 1.83 | 0.00 (-0.16, 0.17) |  | 0.00 (-0.10, 0.10) |  |
|  | Infected with any two helminths (n=155) | GM 1.70 | -0.03 (-0.16, 0.11) |  | -0.02 (-0.11, 0.07) |  |
|  | Infected with any three or four helminths (n=17) | GM 1.75 | -0.02 (-0.24, 0.21) | 0.86 | 0.05 (-0.19, 0.28) | 0.89 |
| Infection with multiple helminth species (testing for trend) |  |  | -0.01 (-0.10, 0.09) | 0.88 | -0.00 (-0.06, 0.05) | 0.89 |
| **Systolic blood pressure (mmHg)** |  |  |  |  |  |  |
| *S. mansoni,* stool KK | Uninfected (n=1065) | 113.82 |  |  |  |  |
|  | Infected (n=440) | 115.38 | 1.56 (-0.22, 3.33) | 0.08 | 0.67 (-1.61, 2.95) | 0.55 |
| *S. mansoni,* stool PCR | Uninfected (n=793) | 113.90 |  |  |  |  |
|  | Infected (n=694) | 114.62 | 0.73(-0.88, 2.33) | 0.36 | 0.47 (-1.36, 2.30) | 0.60 |
| *S. mansoni* intensity, stool KK | Uninfected (n=1065) | 113.82 |  |  |  |  |
|  | Light (n=230) | 115.80 | 1.99 (0.21, 3.76) |  | 1.79 (-0.58, 4.17) |  |
|  | Moderate (n=121) | 115.80 | 1.98 (-2.19, 6.15) |  | 0.01 (-4.03, 4.04) |  |
|  | Heavy (n=89) | 113.72 | -0.10 (-2.84, 2.65) | 0.17 | -1.41 (-3.62, 0.81) | 0.09 |
| *T. trichiura,* stool KK | Uninfected (n=1383) | 114.52 |  |  |  |  |
|  | Infected (n=122) | 111.55 | -2.97 (-6.21, 0.28) | 0.07 | -1.14 (-3.90, 1.62) | 0.40 |
| Hookworm, stool PCR | Uninfected (n=1309) | 114.48 |  |  |  |  |
|  | Infected (n=55) | 109.13 | -5.36 (-9.76, -0.96) | 0.02 | -2.37 (-4.83, 0.09) | 0.06 |
| *Strongyloides stercoralis,* stool PCR | Uninfected (n=1385) | 114.04 |  |  |  |  |
|  | Infected (n=101) | 117.26 | 3.22 (-1.09, 7.53) | 0.14 | 0.19 (-3.15, 3.52) | 0.91 |
| Infection with multiple helminth species (*S. mansoni* [PCR], *T. trichiura*, Hookworm and *S. stercoralis)* | Helminth uninfected (n=655) | 113.89 |  |  |  |  |
|  | Infected with any one helminth (n=611) | 114.91 | 1.02 (-0.59, 2.64) |  | 0.88 (-1.14, 2.89) |  |
|  | Infected with any two helminths (n=155) | 113.32 | -0.58 (-3.52, 2.39) |  | -0.60 (-2.96, 1.75) |  |
|  | Infected with any three or four helminths (n=17) | 109.78 | -4.11 (-9.84, 1.61) | 0.33 | -1.95 (-8.00, 4.11) | 0.55 |
| Infection with multiple helminth species (testing for trend) |  |  | -0.02 (-1.22, 1.17) | 0.97 | 0.01 (-1.13, 1.15) | 0.99 |
| **Diastolic blood pressure (mmHg)** |  |  |  |  |  |  |
| *S. mansoni,* stool KK | Uninfected (n=1065) | 76.30 |  |  |  |  |
|  | Infected (n=440) | 75.86 | -0.44 (-1.89, 1.01) | 0.54 | 0.72 (-1.40, 2.84) | 0.49 |
| *S. mansoni,* stool PCR | Uninfected (n=793) | 76.35 |  |  |  |  |
|  | Infected (n=694) | 75.88 | -0.46 (-1.48, 0.56) | 0.36 | 0.56 (-0.84, 1.95) | 0.42 |
| *S. mansoni* intensity, stool KK | Uninfected (n=1065) | 76.30 |  |  |  |  |
|  | Light (n=230) | 76.58 | 0.27 (-1.08, 1.63) |  | 1.25 (-0.71, 3.22) |  |
|  | Moderate (n=121) | 76.75 | 0.45 (-2.56, 3.47) |  | 1.55 (-2.35, 5.46) |  |
|  | Heavy (n=89) | 72.84 | -3.46 (-4.81, -2.12) | <0.01 | **-2.29 (-3.91, -0.68)** | **0.01** |
| *T. trichiura,* stool KK | Uninfected (n=1383) | 76.40 |  |  |  |  |
|  | Infected (n=122) | 72.97 | -3.43 (-5.64, -1.22) | <0.01 | -1.45 (-3.58, 0.69) | 0.18 |
| Hookworm, stool PCR | Uninfected (n=1309) | 76.35 |  |  |  |  |
|  | Infected (n=55) | 72.44 | -3.90 (-7.22, -0.59) | 0.02 | 2.20 (-0.92, 5.33) | 0.16 |
| *Strongyloides stercoralis, s*tool PCR | Uninfected (n=1385) | 76.09 |  |  |  |  |
|  | Infected (n=101) | 76.60 | 0.51 (-2.67, 3.69) | 0.74 | -0.56 (-3.25, 2.14) | 0.68 |
| Infection with multiple helminth species (*S. mansoni* [PCR], *T. trichiura*, Hookworm and *S. stercoralis)* | Helminth uninfected (n=655) | 76.44 |  |  |  |  |
|  | Infected with any one helminth (n=611) | 76.34 | -0.10 (-1.22, 1.02) |  | 0.87 (-0.80, 2.53) |  |
|  | Infected with any two helminths (n=155) | 74.07 | -2.37 (-4.33, -0.41) |  | 0.08 (-2.21, 2.38) |  |
|  | Infected with any three or four helminths (n=17) | 70.78 | -5.66 (-12.24, 0.91) | 0.10 | -4.11 (-10.33, 2.11) | 0.47 |
| Infection with multiple helminth species (testing for trend) |  |  | -0.94 (-1.70, -0.18) | 0.02 | -0.26 (-1.13, 0.61) | 0.54 |
| **Body mass index (kg/m2)** |  |  |  |  |  |  |
| *S. mansoni,* stool KK | Uninfected (n=1065) | 23.83 |  |  |  |  |
|  | Infected (n=440) | 22.75 | -1.07 (-1.47, -0.67) | <0.01 | -0.02 (-0.53, 0.49) | 0.93 |
| *S. mansoni, s*tool PCR | Uninfected (n=793) | 24.03 |  |  |  |  |
|  | Infected (n=694) | 22.88 | -1.15 (-1.67, -0.63) | <0.01 | -0.35 (-0.93, 0.24) | 0.23 |
| *S. mansoni* intensity, stool KK | Uninfected (n=1065) | 23.83 |  |  |  |  |
|  | Light (n=230) | 23.10 | -0.72 (-1.31, -0.14) |  | 0.12 (-0.45, 0.69) |  |
|  | Moderate (n=121) | 22.44 | -1.39 (-1.90, -0.87) |  | -0.35 (-0.84, 0.14) |  |
|  | Heavy (n=89) | 22.30 | -1.53 (-2.43, -0.62) | <0.01 | 0.07 (-0.83, 0.96) | 0.24 |
| *T. trichiura,* stool KK | Uninfected (n=1383) | 23.57 |  |  |  |  |
|  | Infected (n=122) | 22.28 | -1.29 (-2.48, -0.10) | 0.03 | -0.58 (-1.42, 0.25) | 0.16 |
| Hookworm, stool PCR | Uninfected (n=1309) | 23.53 |  |  |  |  |
|  | Infected (n=55) | 21.73 | -1.80 (-3.36, -0.25) | 0.03 | 0.00 (-1.17, 1.17) | 1.00 |
| *Strongyloides stercoralis,* stool PCR | Uninfected (n=1385) | 23.52 |  |  |  |  |
|  | Infected (n=101) | 22.92 | -0.60 (-1.68, 0.49) | 0.27 | 0.10 (-1.04, 1.24) | 0.87 |
| Infection with multiple helminth species (*S. mansoni* [PCR], *T. trichiura*, Hookworm and *S. stercoralis)* | Helminth uninfected (n=655) | 24.06 |  |  |  |  |
|  | Infected with any one helminth (n=611) | 23.11 | -0.95 (-1.64, -0.26) |  | -0.22 (-0.85, 0.40) |  |
|  | Infected with any two helminths (n=155) | 22.25 | -1.82 (-2.59, -1.04) |  | -0.46 (-1.14, 0.22) |  |
|  | Infected with any three or four helminths (n=17) | 21.03 | -3.03 (-4.49, -1.57) | <0.01 | 0.11 (-1.41, 1.63) | 0.61 |
| Infection with multiple helminth species (testing for trend) |  |  | -0.94 (-1.33, -0.55) | <0.01 | -0.19 (-0.50, 0.12) | 0.21 |
| **Waist circumference (cm)** |  |  |  |  |  |  |
| *S. mansoni,* stool KK | Uninfected (n=1065) | 81.44 |  |  |  |  |
|  | Infected (n=440) | 78.69 | -2.74 (-3.82, -1.67) | <0.01 | -0.27 (-1.92, 1.39) | 0.74 |
| *S. mansoni,* stool PCR | Uninfected (n=793) | 81.79 |  |  |  |  |
|  | Infected (n=694) | 79.17 | -2.62 (-3.69, -1.54) | <0.01 | -0.83 (-2.87, 1.22) | 0.41 |
| *S. mansoni* intensity, stool KK | Uninfected (n=1065) | 81.44 |  |  |  |  |
|  | Light (n=230) | 79.06 | -2.38 (-3.93, -0.83) |  | -0.01 (-1.86, 1.85) |  |
|  | Moderate (n=121) | 78.93 | -2.51 (-4.48, -0.53) |  | -0.97 (-2.95, 1.01) |  |
|  | Heavy (n=89) | 77.44 | -4.00 (-6.07, -1.92) | <0.01 | 0.05 (-2.26, 2.36) | 0.61 |
| *T. trichiura,* stool KK | Uninfected (n=1383) | 80.76 |  |  |  |  |
|  | Infected (n=122) | 77.73 | -3.03 (-5.93, -0.13) | 0.04 | 0.19 (-2.12, 2.51) | 0.87 |
| Hookworm, stool PCR | Uninfected (n=1309) | 80.65 |  |  |  |  |
|  | Infected (n=55) | 76.18 | -4.48 (-7.39, -1.57) | <0.01 | 0.50 (-2.53, 3.53) | 0.74 |
| *Strongyloides stercoralis,* stool PCR | Uninfected (n=1385) | 80.55 |  |  |  |  |
|  | Infected (n=101) | 80.49 | -0.05 (-2.68, 2.57) | 0.97 | 1.02 (-2.20, 4.23) | 0.52 |
| Infection with multiple helminth species (*S. mansoni* [PCR], *T. trichiura*, Hookworm and *S. stercoralis)* | Helminth uninfected (n=655) | 81.88 |  |  |  |  |
|  | Infected with any one helminth (n=611) | 79.44 | -2.45 (-3.74, -1.15) |  | -0.82 (-2.65, 1.00) |  |
|  | Infected with any two helminths (n=155) | 78.85 | -3.04 (-5.18, -0.89) |  | 0.62 (-1.50, 2.74) |  |
|  | Infected with any three or four helminths (n=17) | 74.05 | -7.83 (-11.71, -3.95) | <0.01 | 1.12 (-2.07, 4.32) | 0.32 |
| Infection with multiple helminth species (testing for trend) |  |  | -1.98 (-2.91, -1.04) | <0.01 | -0.04 (-1.03, 0.95) | 0.93 |
| **Waist-hip Ratio** |  |  |  |  |  |  |
| *S. mansoni,* stool KK | Uninfected (n=1065) | 0.85 |  |  |  |  |
|  | Infected (n=440) | 0.85 | 0.00 ( -0.01, 0.01) | 0.85 | 0.00 (-0.01, 0.01) | 0.57 |
| *S. mansoni,* stool PCR | Uninfected (n=793) | 0.85 |  |  |  |  |
|  | Infected (n=694) | 0.85 | 0.00 (-0.01, 0.00) | 0.48 | 0.00 (-0.01, 0.01) | 0.76 |
| *S. mansoni* intensity, stool KK | Uninfected (n=1065) | 0.85 |  |  |  |  |
|  | Light (n=230) | 0.85 | 0.00 (-0.01, 0.01) |  | 0.00 (-0.01, 0.02) |  |
|  | Moderate (n=121) | 0.85 | 0.00 (-0.01, 0.01) |  | 0.00 (-0.01, 0.01) |  |
|  | Heavy (n=89) | 0.85 | 0.00 (-0.01, 0.01) | 0.94 | 0.00 (-0.01, 0.02) | 0.95 |
| *T. trichiura,* stool KK | Uninfected (n=1383) | 0.85 |  |  |  |  |
|  | Infected (n=122) | 0.84 | -0.01 (-0.02, 0.01) | 0.26 | -0.01 (-0.03, 0.01) | 0.3 |
| Hookworm, stool PCR | Uninfected (n=1309) | 0.85 |  |  |  |  |
|  | Infected (n=55) | 0.85 | 0.00 (-0.01, 0.02) | 0.70 | 0.01 (-0.01, 0.03) | 0.38 |
| *Strongyloides stercoralis.* stool PCR | Uninfected (n=1385) | 0.85 |  |  |  |  |
|  | Infected (n=101) | 0.87 | 0.02 (0.01, 0.04) | <0.01 | 0.02 (0.00, 0.03) | 0.04 |
| Infection with multiple helminth species (*S. mansoni* [PCR], *T. trichiura*, Hookworm and *S. stercoralis)* | Helminth uninfected (n=655) | 0.85 |  |  |  |  |
|  | Infected with any one helminth (n=611) | 0.85 | 0.00 (-0.01, 0.00) |  | 0.00 (-0.01, 0.01) |  |
|  | Infected with any two helminths (n=155) | 0.86 | 0.01 (0.00, 0.02) |  | 0.01 (0.00, 0.02) |  |
|  | Infected with any three or four helminths (n=17) | 0.85 | 0.01 (-0.02, 0.03) | 0.11 | 0.01 (-0.01, 0.03) | 0.27 |
| Infection with multiple helminth species (testing for trend) |  |  | 0.00 (0.00, 0.01) | 0.60 | 0.00 (-0.01, 0.01) | 0.59 |

*CI, Confidence intervals; GM, Geometric mean; KK, Kato Katz; PCR, Polymerase Chain Reaction;* **Both crude and adjusted results allowed for the survey design i.e. weighting and clustering.* ***Adjusted for the following variables:* ***HOMA-IR*** *(age, sex, occupation, residence, lake contact, treatment for worms, treatment with coartem, BMI, family history of DM, maternal and paternal tribe);* ***Fasting blood glucose*** *(sex, age, occupation, residence, lake contact, treatment for worms, BMI, maternal tribe);* ***Glycated haemoglobin*** *(age, sex, occupation, residence, lake contact, treatment for worms, paternal tribe, maternal tribe, BMI, family history of diabetes );* ***Triglycerides*** *(age, sex, occupation, residence, diet, treatment for worms, family history of obesity, paternal and maternal tribe);* ***Total cholesterol*** *(age, sex, occupation, residence, diet, exercise, family history of obesity, paternal and maternal tribe);* ***LDL – Cholesterol*** *(age, sex, occupation, diet, exercise, treatment for worms, treatment with coartem, parental tribe );* ***HDL – Cholesterol*** *(age, sex, occupation, residence, diet, treatment for worms, treatment with coartem, parental tribe);* ***Systolic blood pressure*** *(age, sex, occupation, residence, treatment with coartem, maternal tribe, BMI);* ***Diastolic blood pressure*** *(age, sex, occupation, residence, lake contact, treatment for worms, paternal tribe, BMI);* ***Body mass index*** *(age, sex, occupation, residence, lake contact, diet, parental tribe, family history of obesity);* ***Waist circumference*** *(age, sex, occupation, residence, lake contact, diet, treatment for worms, treatment with coartem, parental tribe);* ***Waist-hip Ratio*** *(age, sex, occupation, residence, lake contact, diet, treatment for worms)*
